# Characterization of Model Peptide Adducts with Reactive Metabolites of Naphthalene by Mass Spectrometry

**DOI:** 10.1371/journal.pone.0042053

**Published:** 2012-08-03

**Authors:** Nathalie T. Pham, William T. Jewell, Dexter Morin, A. Daniel Jones, Alan R. Buckpitt

**Affiliations:** 1 Department of Molecular Biosciences, School of Veterinary Medicine, University of California Davis, Davis, California, United States of America; 2 Molecular Structure Facility, University of California Davis, Davis, United States of America; 3 Department of Biochemistry and Molecular Biology and Department of Chemistry, Michigan State University, East Lansing, Michigan, United States of America; University Paris Diderot-Paris 7, France

## Abstract

Naphthalene is a volatile polycyclic aromatic hydrocarbon generated during combustion and is a ubiquitous chemical in the environment. Short term exposures of rodents to air concentrations less than the current OSHA standard yielded necrotic lesions in the airways and nasal epithelium of the mouse, and in the nasal epithelium of the rat. The cytotoxic effects of naphthalene have been correlated with the formation of covalent protein adducts after the generation of reactive metabolites, but there is little information about the specific sites of adduction or on the amino acid targets of these metabolites. To better understand the chemical species produced when naphthalene metabolites react with proteins and peptides, we studied the formation and structure of the resulting adducts from the incubation of model peptides with naphthalene epoxide, naphthalene diol epoxide, 1,2-naphthoquinone, and 1,4-naphthoquinone using high resolution mass spectrometry. Identification of the binding sites, relative rates of depletion of the unadducted peptide, and selectivity of binding to amino acid residues were determined. Adduction occurred on the cysteine, lysine, and histidine residues, and on the N-terminus. Monoadduct formation occurred in 39 of the 48 reactions. In reactions with the naphthoquinones, diadducts were observed, and in one case, a triadduct was detected. The results from this model peptide study will assist in data interpretation from ongoing work to detect peptide adducts *in vivo* as markers of biologic effect.

## Introduction

A large body of evidence supports the concept that electrophiles, generated intracellularly from chemically stable compounds, play a key role in chemical-induced cytotoxicity [1], [2]. Despite positive correlations between total levels of reactive metabolite binding and the extent of toxicity for many chemicals, there are notable exceptions where high levels of bound metabolite do not lead to detrimental effects in the cell [3], [4]. These inconsistencies suggest that simple measures of reactive metabolite formation cannot, a priori, be used to exclude a prospective therapeutic agent from development and that better methods are needed to discriminate those reactive metabolites with real potential for tissue injury from those which are unlikely to cause deleterious effects [5].

Considerable progress has been made in enhancing the ease and speed of studying proteins adducted by reactive metabolites and, owing largely to Hanzlik’s work, a database is now available which lists many of the cellular proteins adducted by reactive intermediates [6]. In addition, biotin-tagged model chemicals that alkylate proteins via S_N_2 mechanisms have also allowed identification of several low abundance proteins which have been covalently modified [7], [8]. However, although hypotheses have emerged on which proteins may be important in the pathways leading to cell death, the identification of many of the critical proteins remains elusive.

Naphthalene is a polycyclic aromatic hydrocarbon present in jet fuel, cigarette smoke, and fossil fuel combustion byproducts, and is used industrially in the production of phthalate plasticizers and dyes [9], [10]. The recent listing of naphthalene as ‘reasonably anticipated to be a human carcinogen’ by the National Toxicology Program is based on dose-dependent tumors observed in rodents during cancer bioassays [11]. However, although naphthalene has been shown to produce species specific toxicity in the respiratory tract of rodents, epidemiological studies cannot discriminate contributions of naphthalene from other airborne toxicants to the incidence of diseases in the human respiratory system. Thus, further tools are needed to better establish the potential human health consequences from naphthalene exposure.

Naphthalene biotransformation involves the generation of multiple reactive metabolites, including epoxides and quinones, which have the potential to bind covalently to amino acid residues [12], [13], [14]. The purpose of this study was to characterize the amino acid residues that react with these reactive metabolites, to determine whether these could be measured effectively in a very controlled environment where the pH and peptide/metabolite ratios could be controlled and to measure relative rates of adduction. Several studies have identified amino acid targets of reactive metabolites from other compounds with abundant proteins like albumin *in vitro* [15], [16], but with the exception of the studies published by Ikehata where specific amino acid residue adduction sites were identified with thiobenzamide, there is relatively little information available about amino acid targets within whole proteins *in vivo* [17].

Two of the six model peptide sequences in this study were from proteins previously identified as adducts of naphthalene. The other sequences were selected based on the presence of putative target residues (cysteine, lysine, and histidine) to aid in the technical evaluation of adduction sites in a controlled environment. Rates of modification of these nucleophilic sites would presumably help guide predictions of side chain reactivity for other candidate proteins.

High resolution tandem mass spectrometry (HR-MS/MS) was used to characterize the adducts. These analyses aimed to establish: 1) the binding site for each metabolite, 2) the reactivity of each peptide in different solution conditions, and 3) the affinity of an electrophilic metabolite for more than one nucleophilic site within the same peptide. As demonstrated recently, even with HR-MS, identifying specific adducted amino acid residues on proteins remains challenging [18]. The work described here is part of an ongoing effort to investigate adducted urinary peptides of naphthalene as a means to compare the formation of critical and non-critical protein adducts through signature MS characteristics. Specifically, this model peptide system has furthered our understanding of relevant MS/MS patterns and made possible identifications of adducting moieties and target amino acids in peptides isolated in urine of naphthalene-treated animals. Future comparisons between urinary peptide adduct patterns in susceptible and non susceptible species and exposed humans could be used to assess potential human health consequences of naphthalene exposure.

## Materials and Methods

### Reagents

Peptides GRGDSPC, DYKDDDDK, Leucokinin IV (DASFHSWG-NH_2_), as well as fragments from α-neo-endorphin (YGGFLRKR), protein disulfide isomerase (EFYAPWCG), and actin (EIVRDIKE) were purchased from the American Peptide Company, Inc (Sunnyvale, CA, USA), stored at −20°C, and used without further purification. All peptides yielded a single UV peak (215 nm) as evaluated by the manufacturer using reversed phase HPLC in two solvent systems. ESI/MS was used to demonstrate that peptides were intact. 1,2-Naphthoquinone (1,2-NQ) and 1,4-naphthoquinone (1,4-NQ) were purchased from Sigma-Aldrich (St. Louis, MO, USA), stored at −80°C, and used without further purification.

### Synthesis of Naphthalene Epoxides

Naphthalene 1,2-epoxide (NO) and naphthalene 1,2-dihydro-1,2-dihydroxynaphthalene-3,4-epoxide (diol epoxide, NDO) were synthesized using methods previously published by Yagi and Jerina and Tsang et al., respectively [19], [20]. NO was recrystallized from ethanol to yield white crystals. The concentration of the product in solution was determined by UV absorbance at 268 nm; absence of significant UV absorbance at 309 nm was evidence that the preparation was not contaminated by the primary rearrangement product, 1-naphthol. NDO was recrystallized from chloroform to yield white crystals. Products were redissolved in 99.5% ethanol/0.5% triethylamine (epoxide) or ethanol (diol epoxide) and stored at −80°C under argon.

### Preparation of Naphthalene Metabolite-peptide Adducts

A solution of stock peptide (1 mg/ml, 1 ml) in 0.1M sodium phosphate buffer (pH 8.5 or 7.4) was incubated with the selected metabolite (0.33M, 33 µl) (epoxides in ethanol, naphthoquinones in dimethyl sulfoxide (DMSO)) in a sealed vial under argon. The final concentration of the metabolite was 9.90 µM, which was an approximate 10-fold molar excess of metabolite to peptide. The vial was stirred on a vortex mixer for 15 s and then mixed by continuous inversion for 1 h at room temperature. The pH was measured at the beginning and the end of each incubation and remained constant. For control incubations, the metabolite was omitted. The reaction was stopped by the addition of 10 µL formic acid (FA). Products were stored at −20°C until further analysis. This procedure was performed with NO, 1,2-NQ, 1,4-NQ, and NDO separately with each of the peptides for a total of 48 incubations.

### Rates of Adduct Formation and Preferential Binding

To study the rate of peptide modification by metabolite, reactions were performed at pH 8.5 (optimal stability of the naphthalene epoxide toward hydrolysis) in 0.1M sodium phosphate buffer. Products from incubations were analyzed by HR-MS/MS. Basic comparisons between epoxides and quinones were drawn using 1,2-naphthalene epoxide and 1,2-naphthoquinone.

Model peptide adducts were prepared as described above to assess adduct formation by measurement of unadducted peptide depletion. After metabolite addition, aliquots of the reaction were removed and quenched with 10 µl FA at the following time points: 30 sec, 1 min, 2 min, 4 min, and 8 min; an aliquot for the positive control time point at 0 min was also taken. Peptide to metabolite ratios of 1∶1 and 1∶10 were investigated. Depletion of unadducted peptide was monitored by selective ion monitoring (SIM). These were conducted for GRGDSPC, DYKDDDDK, and DASFHSWG-NH_2_ with 1,2-naphthalene epoxide and 1,2-naphthoquinone.

To examine preferential binding between the sites of adduction (Cys, Lys, and His) with epoxides and quinones, time course studies were also conducted with all three peptides incubated together with either NO or 1,2-NQ. Aliquots of the reaction were removed and quenched by the addition of FA at the time points: 0 min, 30 sec, 1 min, 2 min, 4 min, and 8 min; aliquots for the control (without metabolite) were taken at each time point.

### High Resolution Mass Spectrometry Analysis

Samples were analyzed with a LTQ Orbitrap (Thermo Fisher, San Jose, CA) operated in positive mode with an IonMax electrospray ionization source. Compounds were separated by HPLC on a 1 x 50 mm Waters Symmetry C_18_ column. Mobile phase A was 0.1% FA/H_2_O and mobile phase B was 0.1% FA acid/acetonitrile (ACN); initial HPLC gradient conditions were 95% A and 5% B at a flow rate 200 µL/min. The gradient was increased linearly from 5% B to 50% B over 25 min, increased to 100% B over the next 3 min, then returned to initial conditions over the last 3 min. The HPLC effluent was monitored in FT mode in the *m*/*z* 200 to 1500 range with a spray voltage of 5.00kV, a capillary temperature of 250°C, and a capillary voltage of 49V. Collision induced dissociation was utilized to fragment the [M+H]^+^ ions. The MS/MS analysis of the peptide adducts was performed with 35% normalized collision energy and the product ions were scanned in the Orbitrap. The resolving power was 30,000 for the full scan MS event and MS/MS events. Data were analyzed with Xcalibur Qual Browser software (Thermo Fisher) and spectra were validated against predicted fragment ions using Protein Prospector (http://prospector.ucsf.edu).

To determine the time course of adduct generation, reactions products were analyzed again as described above with the following modification: instead of a full scan, the protonated peptide adducts were chosen as ions for SIM detection. To look at competitive binding between the amino acid sites, the disappearance of the unadducted peptide was also measured using SIM. Steeper gradients consisting of 95% A and 5% B initially followed by a linear increase to 50% B over 12 min and a final increase to 100% B over the last 3 min were used. Integrations of peptide ion peaks were performed using Xcalibur Qual Browser after optimization of peak integration parameters. Peak detection was by the Genesis algorithm; LC profile trace was by base peak; smoothing was performed using a 15-point smooth, and baselines were subtracted. Although alignment software was not utilized, our samples were relatively pure and SIM was used to focus on target ions in the chromatographic profiles, and the identification and peak integration of peptide ions were unambiguous.

## Results

### MS and MS/MS Analysis of Naphthalene Metabolite-peptide Adducts

**Figure 1:**
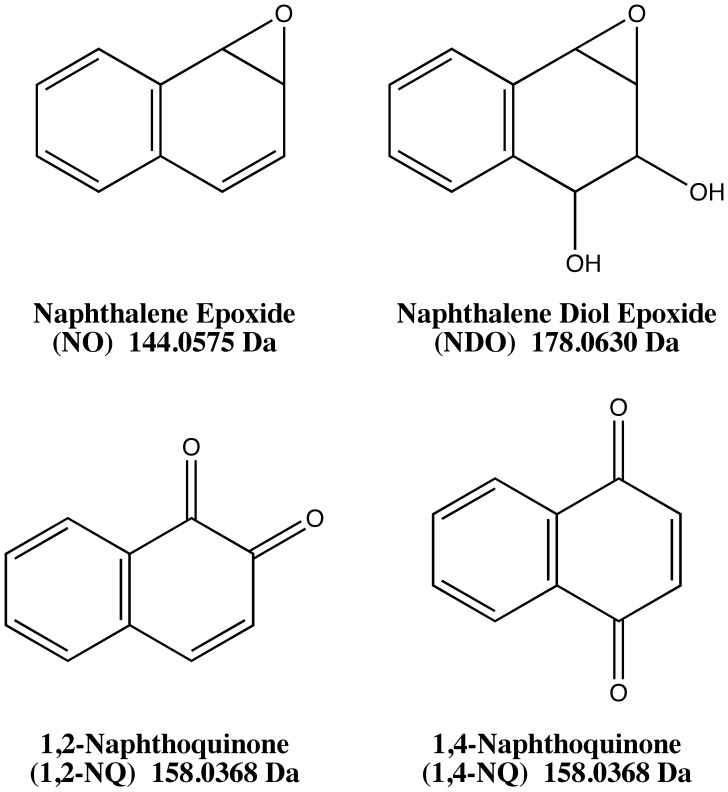
Metabolite structures and peptide sequences. 1A shows the structures of the reactive metabolites of naphthalene. 1B shows the sequences of the model peptides. Sites of potential adduction by reactive metabolites are circled on the amino acid residues of the sequences.

Six peptides (GRGDSPC, YGGFLRKR, DYKDDDDK, DASFHSWG-NH_2_, EFYAPWCG_,_ EIVRDIKE) were incubated with the naphthalene metabolites NO, NDO, 1,2-NQ, and 1,4-NQ at pH 7.4 and 8.5. Metabolite structures are given in Figure 1. Adduct identification was achieved by comparing intact masses of the unadducted peptide with the corresponding adducted product and observing the predicted [M+H]^+^ (monoisotopic mass of unmodified peptide + exact mass of metabolite + H^+^); for NO, the mass of the metabolite was 144.06 Da; for NDO, 178.06 Da; for both the NQs, 158.04 Da (hydroquinone form). Adduct formation was confirmed based on fragment ions in the MS/MS of each adduct. Mass spectral summaries of the b-ion and y-ion series for monoadducts are presented in Tables 1 and 2. The ion series for the diadducts are listed in Table 3. For peptides with adduction at both pH conditions, the product ion spectra were indistinguishable between the two conditions. Masses of the [M+H]^+^ ions of the adducted peptides were found to be in good agreement with the predicted masses (Table S1) and the MS/MS spectra from the reactions are shown in the supplemental data. Nomenclature of fragment ions modified with specific metabolite groups is indicated with the following symbols: ^#^ = NO adduct, ^ψ^ = NDO adduct, ^†^ = 1,2-NQ adduct, and ° = 1,4-NQ adduct.

**Table 1:** MS/MS Ion series of unmodified and adduct-modified model peptides by naphthalene epoxide (NO) and naphthalene diol epoxide (NDO) at pH 8.5.

Peptide	Modification	Precursor *m/z* [M+H]^+^	b/y-ions detected a	SupplementalFigure Ref. b
GRGDSPC	NO c	835.34	386.18 (b_4_), 473.21 (b_5_), **450.17 (y_3_** ^#^ **), 565.20 (y_4_** ^#^ **)**, 691.28 ([M+H]^+^- X d),**817.33 ([M+H]^+^-** **H_2_O)**	1
GRGDSPC	NDO e	869.31	271.15 (b_3_), 386.17 (b_4_), 473.21 (b_5_), 570.26 (b_6_), **656.21 (y_5_** ^ψ^ **), 812.32 (y_6_** ^ψ^ **),**691.28 ([M+H]^+^- X), **851.32 ([M+H]^+^-** **H_2_O)**	Figure 2
GRGDSPC	unmodified	691.28	214.13 (b_2_), 271.15 (b_3_), 386.18 (b_4_), 473.21 (b_5_), 570.26 (b_6_), 306.11 (y_3_),634.26 (y_6_)	1
YGGFLRKR	NO	N/A	Precursor mass was not detected. No fragmentation.	N/A
YGGFLRKR	NDO	1174.64	**872.43 (b_6_** ^ψ^ **), 1000.53 (b_7_** ^ψ^ **),** 776.49 (y_6_), 833.51 (y_7_), 996.57 ([M+H]^+^- X),**1156.62 ([M+H]^+^-** **H_2_O)**	5
YGGFLRKR	unmodified	996.57	538.26 (b_5_), 694.37 (b_6_), 712.38 (b_6_+ H_2_O), 822.46 (b_7_), 840.47 (b_7_+ H_2_O),303.21 (y_2_)	5
DYKDDDDK	NO	1139.45 ([M+H]^+^- H_2_O**)** f	752.27 (b_6_), **648.26 (b_4_** ^#^ **– H_2_O), 763.29 (b_5_** ^#^ **– H_2_O), 878.32** **(b_6_** ^#^ **– H_2_O), 1011.36 (b_7_** ^#^ **),** 377.17 (y_3_), 492.19 (y_4_), 607.22 (y_5_)	2
DYKDDDDK	NDO	1191.47	**1045.36 (b_7_** ^ψ^ **)**, 492.19 (y_4_), 607.22 (y_5_), **913.38 (y_6_** ^ψ^ **), 1076.44 (y_7_** ^ψ^ **),**1013.41 ([M+H]^+^- X), **1173.45 ([M+H]^+^-** **H_2_O)**	6
DYKDDDDK	unmodified	1013.41	407.19 (b_3_), 522.22 (b_4_), 637.25 (b_5_), 752.27 (b_6_), 867.30(b_7_), 377.17 (y_3_),492.19 (y_4_), 607.22 (y_5_), 735.33 (y_6_), 898.38 (y_7_)	2,6
DASFHSWG-NH_2_	NO	1049.45	**702.29 (b_5_** ^#^ **)**, **789.32 (b_6_** ^#^ **),** **975.40 (b_7_** ^#^ **)**, **629.28 (y_4_** ^#^ **),** **776.35 (y_5_** ^#^ **), 863.38 (y_6_** ^#^ **), 934.42 (y_7_** ^#^ **),** 905.39 ([M+H]^+^- X), **1031.43** **([M+H]^+^-** **H_2_O)**	3
DASFHSWG-NH_2_	NDO	1083.45	**736.29 (b_5_** ^ψ^ **), 823.32 (b_6_** ^ψ^ **), 1009.40 (b_7_** ^ψ^ **), 663.29 (y_4_** ^ψ^ **), 810.35 (y_5_** ^ψ^ **),** **897.39 (y_6_** ^ψ^ **),** **968.42 (y_7_** ^ψ^ **),** 905.39 ([M+H]^+^- X), **1065.44 ([M+H]^+^- H_2_O)**	7
DASFHSWG-NH_2_	unmodified	905.39	558.23 (b_5_), 645.26 (b_6_), 831.34 (b_7_), 485.22 (y_4_), 719.33 (y_6_), 790.36 (y_7_)	3,7
EFYAPWCG	NO	1116.50	**606.26 (y_4_** ^#^ **)**, **840.36 (y_6_** ^#^ **),** 972.39 ([M+H]^+^- X), **1098.43([M+H]^+^- H_2_O)**	4
EFYAPWCG	NDO	1150.46	794.35 (b_6_), **640.24 (y_4_** ^ψ^ **)**, **711.28 (y_5_** ^ψ^ **) 874.34 (y_6_** ^ψ^ **)**, **1021.41 (y_7_** ^ψ^ **),** **1132.43 ([M+H]^+^- H_2_O)**	8
EFYAPWCG	unmodified	972.39	511.22(b_4_), 608.27 (b_5_), 794.35 (b_6_), 897.36 (b_7_), 462.18 (y_4_), 533.22 (y_5_),696.28 (y_6_)	4,8
EIVRDIKE	NO	N/A	Precursor mass was not detected. No fragmentation.	N/A
EIVRDIKE	NDO	N/A	Precursor mass was not detected. No fragmentation.	N/A
EIVRDIKE	unmodified	1001.56	498.30 (b_4_),613.33 (b_5_), 854.51 (b_7_), 983.55 ([M+H]^+^ – H_2_O – X)	14

Product ion spectra for adducted peptides were indistinguishable between both pH conditions; more products were observed at pH 8.5 therefore ions series for the products at this pH was given.

a Observed signals assigned as b- or y- ions are listed. The bold b- and y- ions represent ions modified by the naphthalene epoxides.

b Reference to detailed supplemental spectra corresponding to the appropriate ion series.

c The modifications are naphthalene epoxide (mass +144.06 Da).

d In the fragment ion signals, X represents the mass of the adduct.

e The modifications are naphthalene diol epoxide (mass +178.06 Da).

f [DYKDDDDK + NO] was observed in the dehydrated form; fragmentation confirmed the adduct.

**Table 2:** MS/MS Ion series of unmodified and adduct-modified model peptides by 1,2-naphthoquinone (1,2-NQ) and 1,4-naphthoquinone (1,4-NQ) at pH 8.5.

Peptide	Modification	*m/z* [M+H]^+^	b/y-ions detected a	Supplemental Figure Ref. b
GRGDSPC	1,2-NQ c	849.32 d	386.18 (b_4_), 473.21 (b_5_), 570.26 (b_6_), **377.12 (y_2_** ^†^ **)**, **792.30 (y_6_** ^†^ **)**,691.28 ([M+H]^+^- X e), **831.29 ([M+H]^+^-** **H_2_O), 849.32 ([M+H]^+^)**	9
GRGDSPC	1,4-NQ f	849.32	271.15(b_3_), 386.18 (b_4_), 473.21 (b_5_), 570.26 (b_6_), **377.11 (y_2_**°**),** **464.15 (y_3_**°**), 831.30** **([M+H]^+^-** **H_2_O)**	15
GRGDSPC	unmodified	691.28	214.13 (b_2_), 271.15 (b_3_), 386.18 (b_4_), 473.21 (b_5_), 570.26 (b_6_),306.11 (y_3_), 634.26 (y_6_)	1 ^g^
YGGFLRKR	1,2-NQ	1154.61	**583.22 (b_4_** ^†^ **), 852.40 (b_6_** ^†^ **), 980.50 (b_7_** ^†^ **)**, 719.47 (y_5_), 776.49 (y_6_),833.51 (y_7_), 996.57 (M+H]^+^- X), **1136.59 ([M+H]^+^- H_2_O)**	10
YGGFLRKR	1,4-NQ	1154.61	538.26 (b_5_), **583.22 (b_4_**°**), 852.41 (b_6_**°**), 980.50 (b_7_**°**),** 719.47 (y_5_),776.49 (y_6_), 833.51 (y_7_), 996.58 ([M+H]^+^- X), **1136.60 ([M+H]^+^- H_2_O)**	16
YGGFLRKR	unmodified	996.57	538.26 (b_5_), 694.37 (b_6_), 822.46 (b_7_), 303.21 (y_2_)	9,16
DYKDDDDK	1,2-NQ	1171.44	**565.23 (b_3_** ^†^ **), 680.26 (b_4_** ^†^ **), 795.28 (b_5_** ^†^ **), 910.31 (b_6_** ^†^ **),** **1025.34 (b_7_** ^†^ **),** 377.17 (y_3_), 492.19 (y_4_), 607.22 (y_5_), **893.35 (y_6_** ^†^ **),** **1056.40 (y_7_** ^†^ **),** **1153.42 ([M+H]^+^- H_2_O)**	11
DYKDDDDK	1,4-NQ	1171.44	752.27 (b_6_), **565.21 (b_3_**°**), 680.24 (b_4_**°**), 795.27 (b_5_**°**), 910.29 (b_6_**°**),** **1025.32 (b_7_**°**)**, 377.17 (y_3_), 492.19 (y_4_), 607.22 (y_5_), **893.35 (y_6_**°**),** **1056.40 (y_7_**°**), 1153.40 ([M+H]^+^- H_2_O)**	Figure 3
DYKDDDDK	unmodified	1013.41	407.19 (b_3_), 522.22 (b_4_), 637.25 (b_5_), 752.27 (b_6_), 867.30(b_7_),377.17 (y_3_), 492.19 (y_4_), 607.22 (y_5_), 735.33 (y_6_), 898.38 (y_7_)	2,6^ h^
DASFHSWG-NH_2_	1,2-NQ	1063.42	**716.26 (b_5_** ^†^ **), 803.30 (b_6_** ^†^ **),** **989.38 (b_7_** ^†^ **)**, **877.36 (y_6_** ^†^ **),** **948.40 (y_7_** ^†^ **), 1045.41 ([M+H]^+^- H_2_O)**	12
DASFHSWG-NH_2_	1,4-NQ	1063.42	**716.27 (b_5_**°**), 803.29 (b_6_**°**), 989.38 (b_7_**°**)**, **877.36 (y_6_**°**),** **948.39 (y_7_**°**),** **1045.40 ([M+H]^+^- H_2_O)**	17
DASFHSWG-NH_2_	unmodified	905.39	558.23 (b_5_), 645.26 (b_6_), 831.34 (b_7_), 485.22 (y_4_), 719.33 (y_6_), 790.36 (y_7_)	3,7^ i^
EFYAPWCG	1,2-NQ	1130.43	608.27 (b_5_), **620.22 (y_4_** ^†^ **)**, **691.25 (y_5_** ^†^ **), 854.32 (y_6_** ^†^ **)**,**1112.41 ([M+H]^+^- H_2_O)**	13
EFYAPWCG	1,4-NQ	1130.43	**620.22 (y_4_**°**)**, **691.25 (y_5_**°**), 854.32 (y_6_**°**)**, **1055.40 (b_7_**°**)**	18
EFYAPWCG	unmodified	972.39	511.22(b_4_), 608.27 (b_5_), 794.35 (b_6_), 897.36 (b_7_), 462.18 (y_4_),533.22 (y_5_), 696.28 (y_6_)	4,8
EIVRDIKE	1,2-NQ	1159.59	504.27 (y_4_), **656.34 (b_4_** ^†^ **), 771.37 (b_5_** ^†^ **), 1012.55 (b_7_** ^†^ **)**,**1141.58 ([M+H]^+^- H_2_O)**	14
EIVRDIKE	1,4-NQ	1159.59	**500.24 (b_3_**°**),** **771.37 (b_5_**°**), 1141.58 ([M+H]^+^- H_2_O)**	19
EIVRDIKE	unmodified	1001.56	498.30 (b_4_),613.33 (b_5_), 854.51 (b_7_), 983.55 ([M+H]^+^ – H_2_O – X)	14, 19

a Product ion spectra for adducted peptides were indistinguishable between both pH conditions; more products were observed at pH 8.5 therefore ions series for the products at this pH was given. Observed signals assigned as b- or y- ions are listed. The bold b- and y- ions represent the adduct-modified ions by the naphthoquinones.

b Reference to detailed supplemental data spectra corresponding to the appropriate ion series.

c The modifications are 1,2-naphthoquinone (mass +158.04).

d The adducted masses were observed from peptide-metabolite incubations performed at both pHs; spectra obtained from products formed at pH 8.5 are given.

e In the detected fragment ion signals, X represents the mass of the adduct.

f The modifications are 1,4-naphthoquinone (mass +158.04).

g,h,i The spectrum for these unmodified peptides are referenced to in the supplemental figures for the epoxide adducts, not the quinone adducts.

**Table 3:** MS/MS Ion series of diadducted model peptides by naphthoquinones at pH 8.5.

Peptide	Modification	Precursor *m/z*[M+H]^+^	Ions Detected a	Supplemental Figure Ref.^b^
GRGDSPC	1,2-NQ c	1007.35 d	**544.22 (b_4_** ^†^ **)** e, **631.25 (b_5_** ^†^ **), 792.30 (y_6_** ^†^ **), 989.36** **([M+H]^+^ - H_2_O +X** g **)**	9
GRGDSPC	1,4-NQ f	1007.35	**728.29 (b_6_)** e, **639.28 (y_4_), 989.35 ([M+H]^+^ - H_2_O +X)**	15
DYKDDDDK	1,2-NQ	1329.47	**1068.35** **(b_6_** ^††^ **)**, **1183.37** **(b_7_** ^††^ **)**, 607.33 (y_5_), 1013.41 ([M+H]^+^- X),**1311.46 ([M+H]^+^ - H_2_O +X)**	11
DYKDDDDK	1,4-NQ	1329.47	752.27 (b_6_), 867.30 (b_7_), 1013.40 ([M+H]^+^- X)	Not shown
DASFHSWG-NH_2_	1,2-NQ	1221.46	**874.31 (b_5_** ^††^ **), 961.34 (b_6_** ^††^ **), 643.26 (y_4_** ^†^ **), 790. 33 (y_5_** ^†^ **),**905.39 ([M+H]^+^- X), **1203.45 ([M+H]^+^ - H_2_O + X)**	12
DASFHSWG-NH_2_	1,4-NQ	1221.46	**961.34 (b_6_** ^††^ **), 1147.42 (b_7_** ^††^ **), 643.26 (y**_4_^†^ **)**, **790.36 (y_5_** ^†^ **),**905.39 ([M+H]^+^- X), **1203.45 ([M+H]^+^ - H_2_O + X)**	17
EFYAPWCG	1,2-NQ	1288.47	**620.22 (y_4_** ^†^ **)**, **1270.45 ([M+H]^+^ - H_2_O + X)**	13
EFYAPWCG	1,4-NQ	1288.47	794.35 (b_6_)**, 1213.4340 (b_7_** ^††^ **), 778.26 (y_4_** ^††^ **)**, **849.29 (y_5_** ^††^ **),** **1012.36 (y_6_** ^††^ **)**	18

Product ion spectra for adducted peptides were indistinguishable between both pH conditions; more products were observed at pH 8.5 therefore ions series for the products at this pH was given.

a Observed signals assigned as b- or y- ions for the diadducts are listed.

b Reference to detailed spectra corresponding to the appropriate ion series.

c The modifications are 1,2-naphthoquinone (monoadduct mass +158.04).

d The adducted masses were observed in incubations performed at both pHs; data are from pH 8.5 incubations.

e Ions in bold represent modification by a naphthoquinone. A bold b- or y- represents a monoadducted ion. Some of the diadduct MS/MS spectra had monoadduct fragment ions present that were absent in the monoadduct MS/MS spectra.

f The modifications are 1,4-naphthoquinone (monoadduct mass +158.04).

g In the detected fragment ion signals, X represents the mass of the adduct.

### Naphthalene Epoxide (NO)

When incubations were conducted at pH 8.5, a NO monoadduct was observed to form on GRGDSPC, DYKDDDDK, DASFHSWG-NH_2_, and EFYAPWCG (Table 1). Except for DYKDDDDK, adduction sites on the other three peptides were identified. The cysteine of GRGDSPC was believed to be the site of adduction (*m/z* 835, Figure S1). This is supported by the nonadducted b_4_ and b_5_ ions and the adducted y_3_^#^ and y_4_^#^ ions (^#^ indicative of the NO adducted forms) observed in the MS/MS spectrum which provide evidence for adduct formation on one of the three C-terminal residues (SPC), with the cysteine anticipated to be the most reactive nucleophile. Based on the MS/MS, proline cannot be excluded as an adduction site, but because cysteine has been shown in the literature to be a highly nucleophilic site with epoxides, it is unlikely that proline was the preferred site in the monoadduct.

The dehydrated ion [M+H-H_2_O]^+^ at *m/z* 1139 suggested adduct formation for DYKDDDDK (Fig. S2). Several adducted b-ions (particularly b_7_^#^ and dehydrated b_4_^#^ – b_6_^#^) matching theoretical ions one would expect from the product ion spectrum of a NO adduct were observed. Unmodified y- ions were observed and argue for adduction at the N-terminal amino group. Because [b_4_^#^ – H_2_O] (*m/z* 648) was the smallest adducted b-ion observed, the site could not be unambiguously identified.

The ion at *m/z* 1049 was consistent with NO adduction on DASFHSWG-NH_2_ (Fig. S3). Adducted y^#^-ions including and greater than y_4_^#^ as well as adducted b^#^-ions including and greater than b_5_^#^ indicated that adduction was on the histidine. For EFYAPWCG (*m/z* 1116, Fig. S4), evidence for modification was provided by adducted ions, y_4_^#^ and y_6_^#^, which localized the adduct on one of the four C-terminal amino acids (PWCG) with cysteine anticipated to be the most likely site of adduction. For reactions conducted at pH 7.4, NO adducts were only observed for peptides DASFHSWG-NH_2_, DYKDDDDK, and EFYAPWCG.

### Naphthalene Diol Epoxide (NDO)

**Figure 2:**
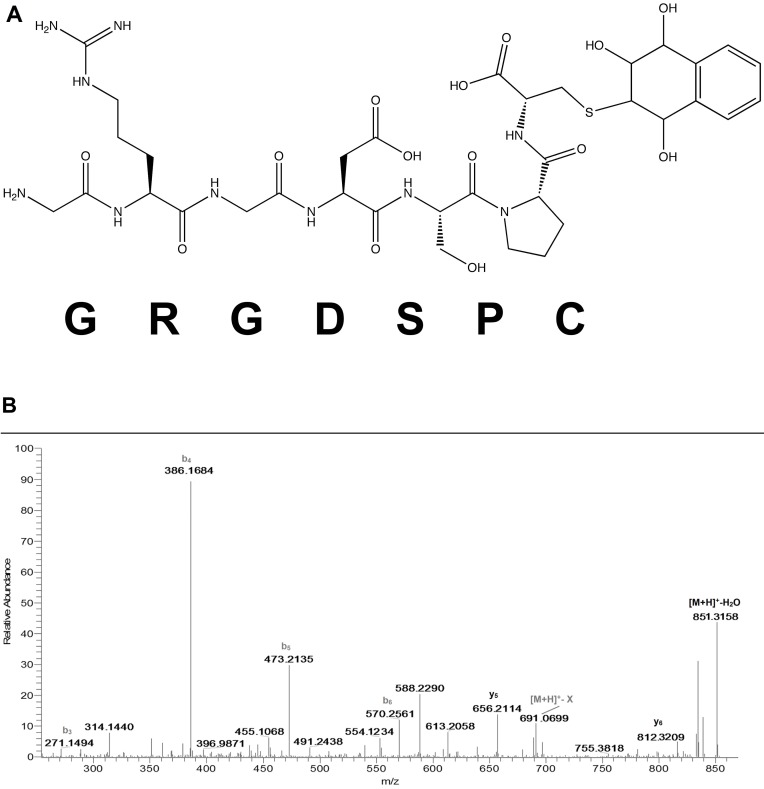
MS/MS for adduction of peptide GRGDSPC by NDO (*m/z* 869.3483). 2A shows the possible y- and b-ions associated with MS fragmentation of the peptide adducted on cysteine. 2B shows the MS/MS spectrum of the adducted peptide acquired with labeled ions for designation. Adducted ions are in bold. Fragmentation patterns were the same when incubations were conducted at either pH 7.4 or 8.5.

At pH 8.5, the NDO adducted peptide masses were observed for all the peptides except for EIVRDIKE (Table 1). For GRGDSPC, the modified peptide was observed as [M+H]^+^ at *m/z* 869 and the MS/MS showed fragment ions (y_5_^ψ^ and y_6_^ψ^) consistent with adduction on cysteine (Figure 2); the lack of adducted b ions supports this assignment. The NDO adduct of YGGFLRKR was observed at *m/z* 1175 (Fig. S5); the modified b_6_^ψ^ and b_7_^ψ^ and unmodified y_6_ and y_7_ ions in the MS/MS of the modified parent ion suggested adduction on the N-terminus. Although the C-terminal Arg is a potential site of adduction, there were no modified y-ions in the spectrum. The DYKDDDDK adduct was observed at *m/z* 1191 (Fig. S6). The lysine at position 3 was believed to be adducted based on the modified b_7_^ψ^, y_6_^ψ^, and y_7_^ψ^ ions (*m/z* 1045, 913, and 1076, respectively). The adducted mass on DASFHSWG-NH_2_ was observed at *m/z* 1083 (Fig. S7); adduction on the histidine was supported by adducted ions: y_4_^ψ^ – y_7_^ψ^ and b_5_^ψ^ – b_7_^ψ^. For EFYAPWCG, the adducted [M+H]^+^ was observed at *m/z* 1150 and supported by adducted ions y_4_^ψ^ - y_7_^ψ^ (Fig. S8). For incubations at pH 7.4, ions consistent with adduct formation were only observed for DASFHSWG-NH_2_, DYKDDDDK, and EFYAPWCG.

### 1,2-Naphthoquinone (1,2-NQ)

1,2-NQ formed adducts with all peptides in incubations conducted at pH 7.4 and 8.5 (Table 2). Diadducts were observed for all reactions conducted at both pH conditions except for YGGFLRKR and EIVRDIKE, which only formed monoadducts (Table 3). The [M+H]^+^ ion at *m/z* 849 in the MS spectrum was consistent with adduct formation on GRGDSPC with support for adduction on one of the two C-terminal residues by adducted ion y_2_^†^ (Fig. S9A). The diadduct of GRGDSPC was observed at *m/z* 1007 and confirmed with monoadducted ions: b_4_^†^, b_5_^†^, and y_6_^†^ (*m/z* 544, 631, and 792) (Table 3, Fig. S9B), consistent with modification on the C-terminal cysteine and N-terminal amino group. A triadduct also was observed for this peptide at *m/z* 1165. MS/MS fragmentation showed the dominant fragment corresponding to neutral loss of two NQ groups and two hydrogen atoms. Monoadducted b_4_^†^ and b_5_^†^, and a diadducted y_6_^††^ suggested that the proline and N-terminal glycine were both adduction sites. For YGGFLRKR, the product ion spectrum of the adducted mass yielded N-terminal fragment ions matching theoretical b-ions as would be expected from a 1,2-NQ monoadduct (Fig. S10); adducted ions b_4_ and b_6_ confirmed adduction at the N-terminal tyrosine. For peptide DYKDDDDK, formation of a monoadduct was supported by [M+H]^+^ *m/z* 1171 (Fig. S11), with adducted N-terminal ions b_3_^†^ – b_7_^†^ and C-terminal ions y_6_^†^ and y_7_^†^, indicating adduction at the lysine at position 3. Fragmentation of the diadduct parent ion at *m/z* 1329 produced diadducted fragment ions b_6_^††^ and b_7_^††^, however the lack of abundant fragments prevented more specific adduct localization; the unmodified y_5_ suggested adduct formation on one of the three N-terminal amino acids. The CID spectrum showed fragment ions consistent with loss of 2 NQ groups plus a hydrogen atom (*m/z* 1013) and the doubly adducted fragment at *m/z* 1068 (b_6_^††^ - H).

The monoadduct and diadduct masses for DASFHSWG-NH_2_ were observed at *m/*z 1063 and *m/z* 1221, respectively (Fig. S12). Histidine was confirmed as the site of adduction based on the adducted b_5_^†^- b_7_^†^, y_6_^†^, and y_7_^†^ ions in the MS/MS of the monoadduct. A comparison of the CID spectra between the monoadduct and diadduct showed a mass shift of 158Da (mass of 1,2-NQ) on the b_5_^†^ and b_6_^†^ ions in the MS/MS of the diadduct, suggesting a second adduction at the N-terminus or diadduct formation on the histidine. Peptide EFYAPWCG formed a monoadduct at *m/z* 1130 and y_4_^†^ – y_6_^†^ (*m/z* 620, 691, and 854, respectively) supported adduction on the cysteine (Fig. S13). No ions corresponding to unmodified N-terminal b-series were observed to provide further supporting evidence. The diadduct mass at *m/z* 1288 was supported by a diadducted y_4_^††^ ion in the MS/MS. EIVRDIKE formed a monoadduct ([M+H]^+^ at *m/z* 1159) and MS/MS fragments suggested the adduct formed on one of the four amino acids closest to the N-terminus based on b_4_^†^, b_5_^†^, and b_7_^†^ fragments (Fig. S14). An unmodified y_7_ supports adduct formation at the N-terminal amino group.

### 1,4-Naphthoquinone (1,4-NQ)

**Figure 3:**
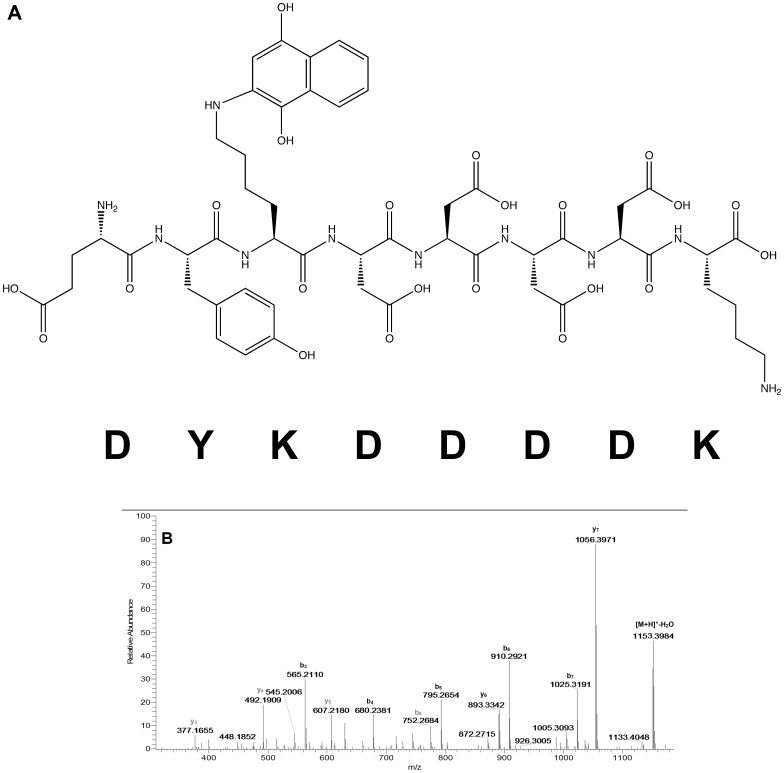
MS/MS for adduction of peptide DYKDDDDK by 1,4-NQ (*m/z* 1171.4366). 3A shows the possible y- and b-ions associated with MS fragmentation of the peptide adducted on lysine. 3B shows the MS/MS spectrum of the adducted peptide acquired with labeled ions for designation. Adducted ions are in bold. Fragmentation patterns were the same when samples were incubated at both pH 7.4 and 8.5.

Adducted masses were observed for all the peptides from reactions conducted at both pHs (Table 2). Diadducts were observed on all peptides except for YGGFLRKR and EIVRDIKE which lack the softer nucleophilic Cys and His side chains (Table 3). An adducted mass for GRGDSPC was observed at *m/z* 849 (Fig. S15). Adducted y-ions (y_2_° and y_3_°) and an absence of adducted b-ions established cysteine as the adduction site. A diadduct mass was observed at *m/z* 1007, but with the exception of b_6_°, there was a lack of adducted ions which precludes precise assignment of the site of adduction for the 1,4-NQ. The YGGFLRKR adduct was observed at *m/z* 1155 (Fig. S16) and modified b-ions (b_4_°, b_6_°, and b_7_°) along with unmodified y-ions (y_5_–y_7_) supported adduction at the N-terminus. The adducted mass for DYKDDDDK at *m/z* 1171 was supported by adducted ions b_3_° – b_7_° and y_6_° and y_7_°, indicating adduction on the lysine at position 3 (Figure 3). The mass of the diadduct was observed at *m/z* 1327 (not shown). The mass of the DASFHSWG-NH_2_ adduct was observed at *m/z* 1063 (Fig. S17); based on the b_5_°-b_7_° and y_6_° and y_7_° ions_,_ the adduction site was the histidine. An ion corresponding to a diadduct (*m/z* 1221) was observed and confirmed by monoadducted ions y_4_° and y_5_°, and diadducted ions b_6_°° and b_7_°°. Peptide EFYAPWCG yielded a monoadduct with 1,4-NQ at *m/z* 1130 and adduction onto the cysteine was confirmed by ions y_4_° – y_6_° and b_7_° (Fig. S18). The diadduct mass was observed at *m/z* 1288 and confirmed by diadducted ions y_4_°° – y_6_°° and b_7_°°, suggestive of two adduct groups on Cys. The mass of EIVRDIKE adduct was observed at *m/z* 1160. The lack of detectable y-ions precluded definitive determination of the adduction site, but the CID spectrum revealed adducted ions b_3_° and b_5_° (Fig. S19), which argues for adduction at the N-terminus.

### Rates of Adduct Formation with Naphthalene Epoxide and Naphthoquinone

Qualitative measurements of the rates of adduct formation were made by monitoring the change in peak area for each unadducted peptide ion over time. Peptides investigated were GRGDSPC, DYKDDDDK, and DASFHSWG-NH_2_ and these showed linear responses within the range of concentrations used in the incubations (Fig S20); standards were diluted to obtain on scale signals. Control incubations measured unadducted peptide concentrations in the absence of metabolites and these data showed a slight loss of GRGDSPC over 8 min (∼15% decrease), while DASFHSWG-NH_2_ and DYKDDDDK remained relatively the same, with a decrease of about 3.6% from the original area during the 8 min incubation (Fig. S21).

**Figure 4:**
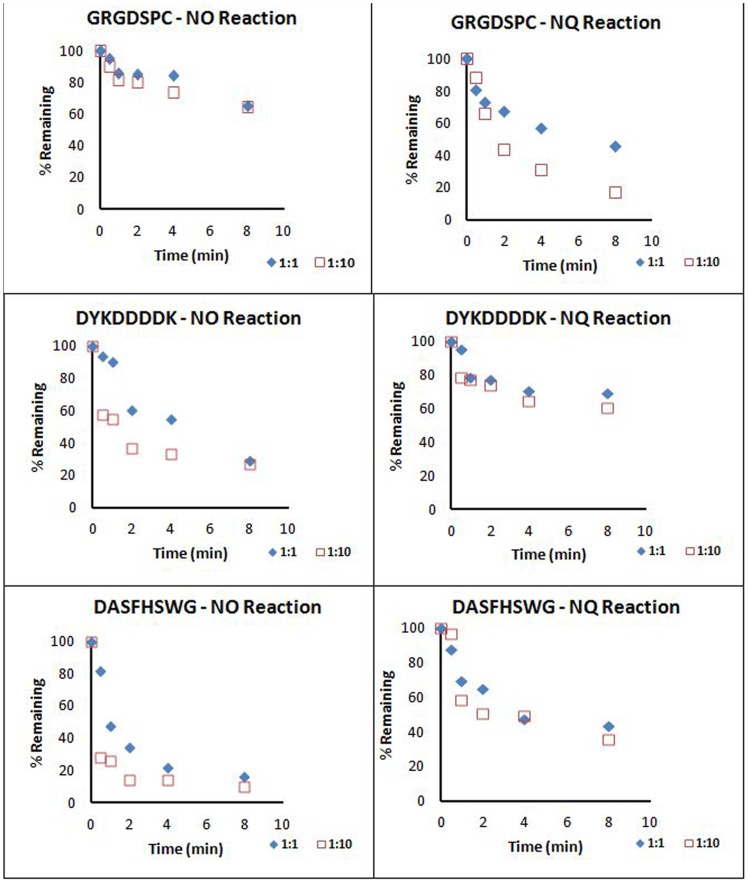
Depletion of unadducted peptides by metabolites. Depletion of GRGDSPC (A), DYKDDDDK (B), and DASFHSWG-NH_2_ (C) by reaction with either NO (naphthalene epoxide) or NQ (1,2-naphthoquinone) at two different ratios of peptide: metabolite, 1∶1 and 1∶10. Depletion of the unadducted peptide was monitored by measuring the peak area of the peptide at each time point.

For GRGDSPC, where cysteine was the target, the 1∶1 and 1∶10 (peptide: metabolite) incubations with NO resulted in very similar losses of unadducted peptide after 8 min (Fig. 4A). The 1∶10 incubation had a faster rate of peptide depletion overall, but by 8 min, both incubations had approximately 70% of unadducted peptide remaining. As stated above, approximately 15% of GRGDSPC was lost in control incubations. In comparison, there was a significant difference in signal loss from the unadducted peptide between the 1∶1 and 1∶10 peptide:metabolite incubations with NQ. After 8 minutes, while the unadducted peptide ion in the 1∶1 incubation had decreased to about 40% of the original area, the ion in the 1∶10 incubation had decreased to less than 20%. The hydrolysis of NQ may have accounted for this slowing in rate.

For DYKDDDDK, where lysine was the target residue, the 1∶10 peptide:metabolite incubation with NO showed a steep initial decrease in peptide signal, but at the 8 min time point approximately the same percent peptide remained as in the 1∶1 incubation, (30%, Fig. 4B). Approximately 60% of the unadducted peptide remained following 8 min incubations with NQ with both 1∶1 and 1∶10 peptide:metabolite ratios.

The loss of the unadducted peptide, DASFHSWG-NH_2_ (where histidine is the target), was similar to that observed with DYKDDDDK. At 8 min, the concentration of unadducted peptide remaining was similar in incubations conducted at 1∶1 and 1∶10 ratios of peptide to NO (Fig. 4B), even though the 1∶10 incubation with NO showed a steeper initial decrease. When incubations were done with NQ at either 1∶1 or 1∶10 peptide:metabolite, peak areas for the unadducted peptide decreased rapidly leaving about 40% of the peptide after 8 minutes.

Adduct formation was also assessed for the incubations by monitoring the change in peak area of adducted ion over time, as shown in Fig. S22.

### Preferential Binding to Different Nucleophilic Sites

**Figure 5:**
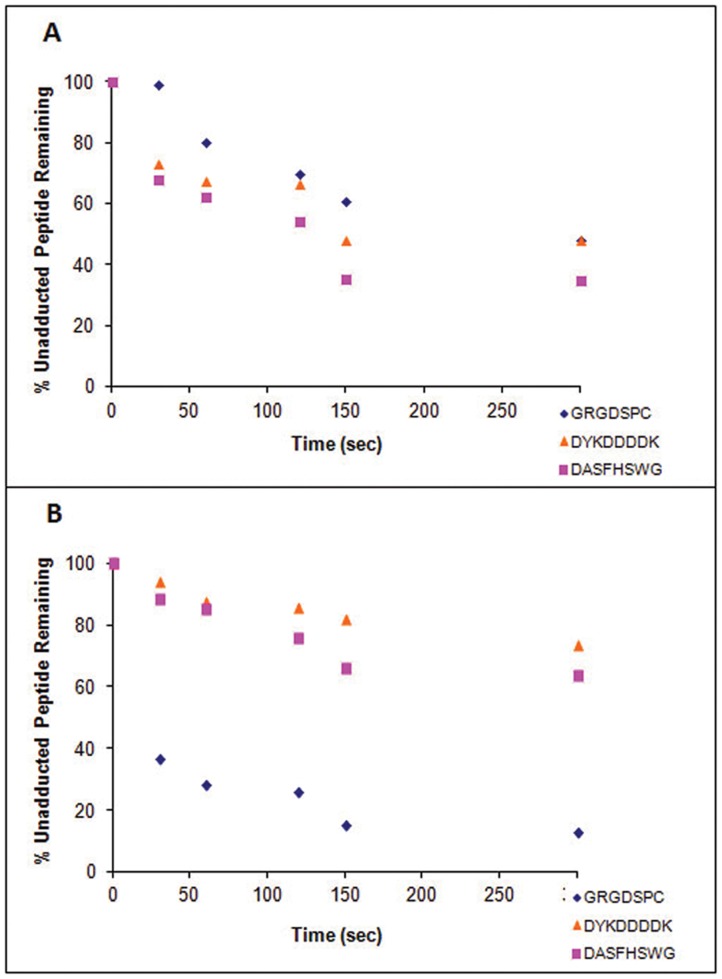
Comparison of peptide binding by metabolite. Comparison of preferential binding between peptides containing different nucleophilic residues (Cys, Lys, and His) in incubations with NO (A) and NQ (B). Degree of preferential binding was monitored by measuring the peak area of the unadducted peptide ion over time.

To assess whether naphthalene reactive metabolites would preferentially adduct different residues, metabolite was added to incubations containing equal concentrations of the model peptides containing a Cys, Lys, or His. Following 8 min incubation with NO, the amount remaining for each unadducted peptide was similar with 40–45% of the peptide remaining. The rate of loss of the lysine (DYKDDDDK) and histidine (DASFHSWG-NH_2_) peptides was initially much faster than the decrease of the cysteine (GRGDSPC) peptide (Fig. 5A).

Adduction of the cysteine containing peptide was highly favored in the NQ incubation. While the histidine and lysine containing unadducted peptides were only depleted to about 70% of the initial level, the cysteine containing peptide was depleted to less than 20% over the same time period (Fig. 5B).

## Discussion

Much of the current insight regarding the importance of electrophile adduction of proteins in cytotoxicity comes from work on compounds where a single reactive metabolite is generated. In cases where multiple reactive metabolites are produced, our understanding of the relative rates of these processes is far more limited. This is the case with naphthalene where at least four reactive metabolites are generated, including the 1,2-epoxide, the diol epoxide, and both the 1,2- and 1,4-naphthoquinones, but little knowledge of the contribution of these metabolites to the levels of covalent adduct formation exists. If the differences in these metabolites’ reactivities with protein residues translate into disparate effects on whether a particular adduct alters the structural or functional properties of a protein critical to cellular homeostasis, then the overall metabolic disposition of this chemical and inter-individual differences in the enzymes responsible for controlling the formation of these metabolites could substantially influence susceptibility to exposure. Thus, the ultimate goal is to assess the impact of electrophile-derived covalent adducts on protein structure and function. The first step toward addressing this larger question was to identify residues of reactive metabolite modification and to determine the preference for the epoxide and quinone metabolites on amino acid residues.

**Figure 6:**
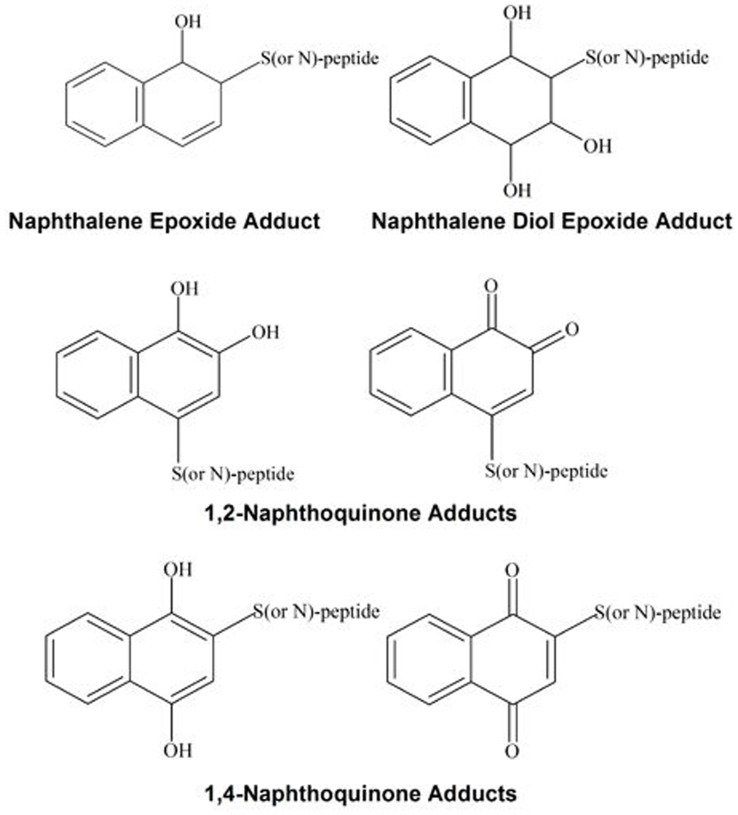
Structures of the predicted adducts. The products of adduct formation with epoxides were the result of S_N_2 reactions while the products of adducts formed with naphthoquinones were the result of Michael Addition reactions.

ESI-MS/MS was used to identify the predicted amino acid targets of naphthalene reactive intermediates (Fig. 6). All the peptides selected contained at least one nucleophilic amino acid shown previously to be adducted by epoxides and/or quinones [21], [22], [23]. The peptides were of high purity facilitating spectral interpretation. The truncated sequences containing the adduction sites (DSPC, LRKR, DYKD, DDDK, and FHSW) were found widely in the UniProt database *(* www.uniprot.org) within biological proteins; DSPC (i.e., P450 2B19, cell division kinase), LRKR (i.e., tyrosine protein kinase, myosin), DYKD (i.e., zinc phosphodiesterase, ubiquitin ligase), DDDK (i.e., serine phosphatase, EGF receptor substrate), and FHSW (i.e., kallikrein, cyclin-dependent kinase). The other two peptides (EFYAPWCG and EIVRDIKE) were from known naphthalene protein targets: actin and protein disulfide isomerase.

It is important to note that electrophilic metabolites do not react with all nucleophilic residues; rather, such interactions occur along a spectrum of reactivity [24]. The softness of a nucleophile is determined by the polarizability of its corresponding valence electrons: the higher the polarizability, the softer the nucleophile. Reactions between electrophiles and nucleophiles of similar softness are kinetically favored because the high energy transition state attained when sharing similar molecular orbitals requires less energy input than if they are dissimilar [25]. This was consistently observed between the naphthoquinones and amines and also contributes to the decreased likelihood of an epoxide to adduct onto the peptide a second time at the N-terminus.

In general, the thiolate of the cysteine is the softest nucleophile in proteins, which makes it the most likely target for the relatively soft naphthalene metabolite electrophiles [12]. Of the two peptides containing cysteine (GRGDSPC and EFYAPWCG), adduction occurred on both with all metabolites at pH 8.5 (Table 4). Whereas adduction was observed between the quinones and GRGDSPC at pH 7.4, no product was detected with the epoxides at pH 7.4. This is likely due to a combination of the reduced amounts of thiolate ion present, the relative reactivity of the quinones compared to the epoxides, and the higher instability of the epoxides at pH 7.4. At pH 7.4, the 1,2-epoxide more rapidly rearranges to 1-naphthol leading to a lower sustained concentration of epoxide at physiological pH. A low pK_a_ also correlates to a higher availability of the thiolate anion at pH 7.4 and a lower overall nucleophilicity due to increased thiolate stabilization from electrostatic and polar interactions from the surrounding environment [26]. Because naphthoquinones are more reactive, they compensate for the decreased nucleophilicity of the thiolate. From the rate of formation study at pH 8.5, there was a much faster depletion of GRGDSPC with NQ compared to NO (Fig. 4A). NQ also reacted more rapidly with the cysteine-containing peptide as compared to either the histidine or lysine containing peptides (Fig. 5B).

**Table 4:** Sites of Adduction.

	pH	
Metabolite	7.4	8.5
**NO**	DYKDDDDK*, DASF**H**SWG-NH_2_, EFYAPW**C**G	GRGDSP**C**, DYKDDDDK*, DASF**H**SWG, EFYAPWC*G
**NDO**	DY**K**DDDDK, DASF**H**SWG-NH_2_, EFYAPW**C**G	GRGDSP**C**, **N-**YGGFLRKR, DY**K**DDDDK, DASF**H**SWG-NH_2_, EFYAPW**C**G
**1,2NQ**	GRGDSP**C**, **N-**YGGFLRKR, DY**K**DDDDK, DASF**H**SWG-NH_2_, EFYAPW**C**G, **N**-EIVRDIKE	GRGDSP**C**, **N-**YGGFLRKR, DY**K**DDDDK, DASF**H**SWG-NH_2_, EFYAPW**C**G, **N**-EIVRDIKE
**1,4NQ**	GRGDSP**C**, **N-**YGGFLRKR, DY**K**DDDDK, DASF**H**SWG-NH_2_, EFYAPW**C**G, **N**-EIVRDIKE	GRGDSP**C**, **N-**YGGFLRKR, DY**K**DDDDK, DASF**H**SWG-NH_2_, EFYAPW**C**G, **N**-EIVRDIKE

Sites of adduction deduced for each peptide.

* represents an undetermined site of adduction. Diadducts and triadduct formed during reactions conducted at pH 8.5 were also formed at pH 7.4.

Adduction was observed on the histidine of DASFHSWG-NH_2_ at both pH 7.4 and 8.5 for all metabolites. The adduct stability is likely due to the imidazole moiety being a poor leaving group. The proximal aspartic acid at the N-terminus may have participated with the histidine in a catalytic triad making adduction more feasible [27]. Another explanation is the aspartate acting as an intramolecular base, enhancing removal of a proton from the His side chain.

Reactivity with the lysine-containing peptides varied. Whether the lack of NO adduct formation on EIVRDIKE and YGGFLRKR was related to a low reaction rate of the epoxide with the peptides or whether the adducted peptides could not be readily detected in the mass spectrometer was unclear. The absence of NO adducts is partially supported by the direct influence of a protein’s microenvironment where it has been shown that pK_a_ plays the dominant role in determining the reactivity of Lys side chains towards electrophiles [28]. In YGGFLRKR, the lysine was flanked by two adjacent arginines (pK_a_ = 12.5), while in EIVRDIKE, the lysine residue was next to glutamic acid (pK_a_ = 4.8). Charges from either highly acidic or highly basic neighbors can influence an adduction. With YGGFLRKR, the presence of the arginines may have decreased the reactivity of the Lys side chain. Likely because of this, a Lys adduction was not kinetically favored and binding occurred preferentially at the N-terminus for YGGLFRKR with the other three metabolites. With EIVRDIKE, adduction by the naphthoquinones did not favor Lys either. With DYKDDDDK, adduction was observed under all conditions. Because the only major difference between this peptide and the others were flanking amino acids, this further validates the significant contribution of the microenvironment to the occurrence of an adduction.

Our studies suggest that the naphthoquinones are more reactive than the epoxides based on the range of detectable adducts as well as the presence of diadducts and a triadduct. Half of the peptides investigated did not react with either epoxide when reactions were done at physiologic pH; adducted products were observed with both naphthoquinones for all peptides when incubated at pH 7.4 and 8.5. The presence of the metabolites in large excess to the peptides was expected to enhance the likelihood of diadduct and triadduct formation, but multiple adductions on the same peptide were not detected with the epoxides, possibly due to a difference in the thermodynamic “softness” as mentioned previously [24]. The results of the current study are consistent with previously published work suggesting that the naphthoquinones are more reactive with proteins than the epoxide metabolites [29].

The time course disappearance of the unadducted peptide was measured in incubations containing 1∶1 and 1∶10 ratios of the peptide to metabolite to obtain an appreciation of the relative rates of reaction of epoxides and quinones with the various nucleophilic sites. LC-MS with SIM was utilized to provide high selectivity for the distinction of peptide peaks from background noise and neighboring peaks, which allowed direct comparison of peptide peak areas between different runs. The initial rates of reaction of NO with both DASFHSWG-NH_2_ and DYKDDDDK had faster depletions in incubations containing a 1∶10 ratio of peptide to metabolite than with those containing a 1∶1 ratio. After 8 minutes, the unadducted peptides were almost completely depleted at both peptide: metabolite ratios, suggesting that the epoxide had saturated the available sites (Fig. 4B and C). No significant differences in the rates of unadducted peptide loss were observed in incubations of 1,2-NQ with these peptides at either the 1∶1 or 1∶10 peptide: metabolite ratios. Only about 50% of the peptides were lost during the 8 min incubation and this may reflect the relative instability of 1,2-NQ in aqueous solutions. Another explanation could be that the NQ formed initially are in the reduced form and can be oxidized by unreacted NQ, leading to depletion of free NQ and yielding quinonoid adducts.

Most of the studies outlined here were performed using peptide to metabolite ratios (1∶10) that are likely to be higher than those observed in target cells *in vivo*. Crude estimates based on the overall formation of adducts in incubations of target tissue subcompartments have yielded overall reactive metabolite binding in the range of 1–4 nmoles/mg protein, depending on the tissue [30]. If the average protein mass of 50 kDa is assumed, this would yield specific activities of between 0.05 and 0.2 nmoles reactive metabolite bound/nmole protein, far lower than the ratios used here. However, our earlier work has shown that there is a reasonable degree of selectivity in adduct formation and so the specific activity of adducted proteins is likely to be much greater than the 0.05 to 0.2 nmoles/mg calculated above [31]. Moreover, as shown by the data in Figure 4, the ratios of reactants did not strongly influence the rates of reaction when peptide: metabolite ratios were increased from 1∶1 to 1∶10. Finally, more recent studies monitoring adducted proteins in the urine [32] indicated that the specific activities of adducted proteins in the urine is 5–10 fold higher than observed in target tissues.

In experiments to evaluate possible competition which might occur when multiple nucleophilic amino acids were present in a mixture, model peptides which contained cysteine, lysine or histidine residues were reacted with NO or 1,2-NQ. Although some differences in rates of loss of unmodified peptide were noted particularly between 1,2-NQ and the cysteine-containing peptide compared to the lysine- and histidine-containing peptides, these experiments likely do not recapitulate the cellular milieu where the secondary and tertiary structure of proteins and proximity to hydrophobic amino acids and/or to basic amino acids such as arginine may dramatically alter reaction rates with electrophiles [26]. Indeed, recent work with protein disulfide isomerase and actin demonstrate relatively high selectivity for modification of specific cysteines, lysines and histidines by both epoxide and quinone metabolites of naphthalene [33].

The results of the current studies are consistent with previous findings showing epoxide binding to sulfur nucleophiles was minor relative to binding by the 1,2-naphthoquinone in Clara cell incubations with naphthalene [34]. In naphthalene-treated mice, 1,4-NQ adducts were more abundant than NO adducts in all tissue samples studied, with the lung carrying the highest NQ load [35]. In contrast, Waidyanatha and Rappaport showed NO- albumin and hemoglobin adducts were more abundant than NQ adducts *in vivo* [12], [36]. Therefore, the relative amounts of metabolite bound covalently to proteins *in vivo* are dependent upon concentrations formed intracellularly, on the availability of cellular protein targets, as well as on the detoxification of reactive intermediates such as NO hydrolysis and GSH conjugation. Although the current work confirmed the target amino acids, additional studies are necessary to determine the relative proportions of epoxides, diol epoxides, and quinones bound to proteins *in vivo*.

This current work characterized both epoxide and quinone adducts with model peptides and these data have been critical to the interpretation of adducted proteins and peptides eliminated in the urine of naphthalene-treated animals in ongoing studies in the laboratory. Understanding the reaction of naphthalene metabolites with biological molecules is critical to delineating the mechanisms by which this chemical leads to the loss of cellular homeostasis. However, rapid adduct formation with a given residue does not imply inherent toxicological significance. Rather, it is the role of the residue in protein structure or function and the resulting disruptive consequences of adduction that determine the relevance of an adduct. Accordingly, additional work which focuses on verifying the sites of adduction *in vivo* as well as investigation into the functional consequences of adduction is underway.

### Supporting Information Available

MS/MS spectra of model peptide adduct products not already shown as figures are presented with corresponding b- and y- ions. Each figure presents either a spectral comparison between the nonadducted parent and the monoadduct, the monoadduct and the diadduct, or the nonadducted parent, the monoadduct, and the diadduct. The *m/z* of the parent peptide is given along with the corresponding MS/MS fragmentation. Adducted ions (modified by a metabolite) are labeled in **bold**, nonadducted ions are not. [M+H]^+^ is representative of the nonadducted parent peptide; **[M+H]^+^** is representative of the monoadducted parent peptide. # is representative of modification by naphthalene epoxide (NO). ψ is representative of modification by naphthalene diol epoxide (NDO). † is representative of modification of 1,2-naphthoqinone (1,2NQ). ° is representative of modification by 1,4-naphthoquinone (1,4NQ). Although spectra were acquired for both pHs, because spectra were closely similar, only spectra acquired at pH 8.5 are given.

Other supporting figures include standard curves and rate of formation. A table presenting mass errors (ppm) is also provided.

## Supporting Information

Figure S1 MS/MS of peptide [GRGDSPC] at *m/z* 691.2826 **(A)** and adduct [GRGDSPC + NO] at *m/z* 835.3408 **(B)**.
(DOCX)

Figure S2 MS/MS of peptide [DYKDDDDK] at *m/z* 1013.4058 **(A)** and adduct [DYKDDDDK + NO] – H_2_O at *m/z* 1139.4510 **(B)**.
(DOCX)

Figure S3 MS/MS of peptide [DASFHSWG-NH_2_] at *m/z* 905.3878 **(A)** and adduct [DASFHSWG-NH_2_+ NO] at *m/z* 1049.4487 **(B)**.
(DOCX)

Figure S4 MS/MS of peptide [EFYAPWCG] at *m/z* 972.3920 **(A)** and adduct [EFYAPWCG + NO] at *m/z* 1116.4995 **(B)**.
(DOCX)

Figure S5 MS/MS of peptide [YGGFLRKR] at *m/z* 996.5738 **(A)** and adduct [YGGFLRKR + NDO] at *m/z* 1174.6365 **(B)**.
(DOCX)

Figure S6 MS/MS of peptide [DYKDDDDK] at *m/z* 1013.4058 **(A)** and adduct [DYKDDDDK + NDO] at *m/z* 1191.4680 **(B)**.
(DOCX)

Figure S7 MS/MS of peptide [DASFHSWG-NH_2_] at *m/z* 905.3900 **(A)** and adduct [DASFHSWG-NH_2_+ NDO] at *m/z* 1083.4537 **(B)**.
(DOCX)

Figure S8 MS/MS of peptide [EFYAPWCG] at *m/z* 972.3920 **(A)** and adduct [EFYAPWCG + NDO] at *m/z* 1150.4550 **(B)**.
(DOCX)

Figure S9 MS/MS of peptide [GRGDSPC +1,2NQ] monoadduct at *m/z* 849.3181 **(A)** and [GRGDSPC +1,2NQ] diadduct *m/z* 1007.3465 **(B)** and [GRGDSPC +1,2NQ] triadduct *m/z* 1165.3826 **(C)**.
(DOCX)

Figure S10 MS/MS of peptide [YGGFLRKR] at *m/z* 996.5738 **(A)** and adduct [YGGFLRKR +1,2NQ] at *m/z* 1154.6139 **(B)**.
(DOCX)

Figure S11 MS/MS of peptide [DYKDDDDK +1,2NQ] monoadduct at *m/z* 1171.4382 **(A)** and [DYKDDDDK +1,2NQ] diadduct at *m/z* 1329.4729 **(B)**.
(DOCX)

Figure S12 MS/MS of peptide [DASFHSWG-NH_2_+1,2NQ] monoadduct at *m/z* 1063.4248 **(A)** and [DASFHSWG-NH_2_+1,2NQ] diadduct at *m/z* 1221.4593 **(B)**.
(DOCX)

Figure S13 MS/MS of peptide [EFYAPWCG +1,2NQ] monoadduct at *m/z* 1130.4287 **(A)** and [EFYAPWCG +1,2NQ] diadduct at *m/z* 1288.4655 **(B)**.
(DOCX)

Figure S14 MS/MS of peptide [EIVRDIKE] at *m/z* 1001.5626 **(A)** and adduct [EIVRDIKE +1,2NQ] at *m/z* 1159.5993 **(B)**.
(DOCX)

Figure S15 MS/MS of peptide [GRGDSPC +1,4NQ] monoadduct at *m/z* 849.3167 **(A)** and [GRGDSPC +1,4NQ] diadduct at *m/z* 1007.3466 **(B)**.
(DOCX)

Figure S16 MS/MS of peptide [YGGFLRKR] at *m/z* 996.5738 **(A)** and adduct [YGGFLRKR +1,4NQ] at *m/z* 1154.6133 **(B)**.
(DOCX)

Figure S17 MS/MS of peptide [DASFHSWG +1,4NQ] monoadduct at *m/z* 1063.4237 **(A)** and [DASFHSWG +1,4NQ] diadduct at *m/z* 1221.4593 **(B)**.
(DOCX)

Figure S18 MS/MS of peptide [EFYAPWCG +1,4NQ] monoadduct at *m/z* 1130.4287 **(A)** and [EFYAPWCG +1,4NQ] diadduct at *m/z* 1288.4655 **(B)**.
(DOCX)

Figure S19 MS/MS of peptide [EIVRDIKE] at *m/z* 1001.5626 **(A)** and adduct [EIVRDIKE +1,4NQ] at *m/z* 1159.5993 **(B)**.
(DOCX)

Figure S20 Plots of areas for [M + H]^+^ ions vs. concentration using selected ion monitoring for nonadducted parent peptide. Different dilutions of unadducted peptide were prepared as described in materials and methods.
(DOC)

Figure S21 Ion monitoring of depletion of unadducted control ([M+H]^ +^) peptide over time; measurement of peak ion area.
(DOC)

Figure S22 Rate of adduct formation for individual incubations for GRGDSPC **(A)**, DASFHSWG **(B)**, and DYKDDDDK **(C)**.
(DOC)

Table S1 PPM errors of adduct ions observed.
(DOC)
