# Supplementary figures and images for: Characterization of Model Peptide Adducts with Reactive Metabolites of Naphthalene by Mass Spectrometry
Source: PLoS One. 2012 Aug 3;7(8):e42053. doi: 10.1371/journal.pone.0042053 (PMC3411726; doi:10.1371/journal.pone.0042053)

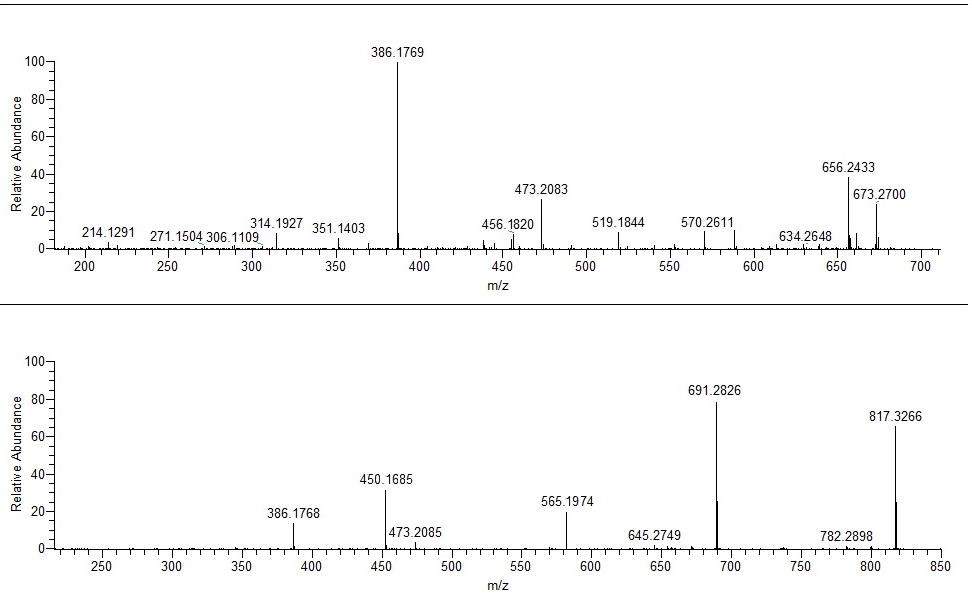


[M+H-NO]+

GRGDSPC

GRGDSPC#

b6

b5

b4

**y4**

**y3**

**[M+H]+ - H2O**

y6

b2

b4

b3

y3

b5

**B**

**A**

**B**

Supplement: Figure S1 — MS/MS of peptide [GRGDSPC] at m/z 691.2826 (A) and adduct [GRGDSPC + NO] at m/z 835.3408 (B). (DOCX) [file pone.0042053.s001.docx]

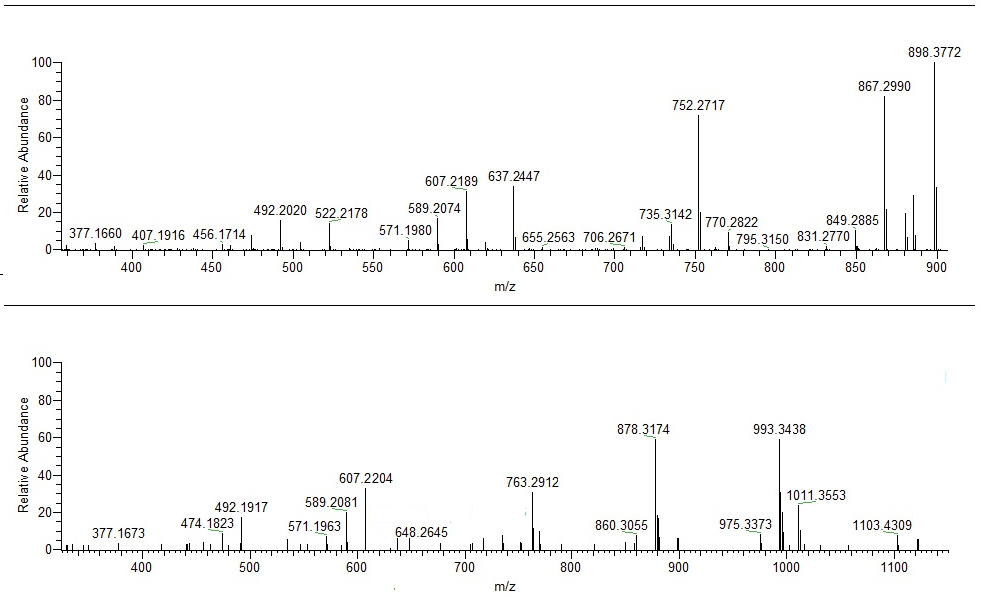


**b6– H2O H2O**

**b7 – H2O**

DYK#(?)DDDDK

DYKDDDDK

**b4 – H2O**

y7

b7

b3

y4

b4

b6

b5

y5

y6

y3

**b7**

**b5– H2O**

y3

y4

y5

**B**

**A**

Supplement: Figure S2 — MS/MS of peptide [DYKDDDDK] at m/z 1013.4058 (A) and adduct [DYKDDDDK + NO] – H2O at m/z 1139.4510 (B). (DOCX) [file pone.0042053.s002.docx]

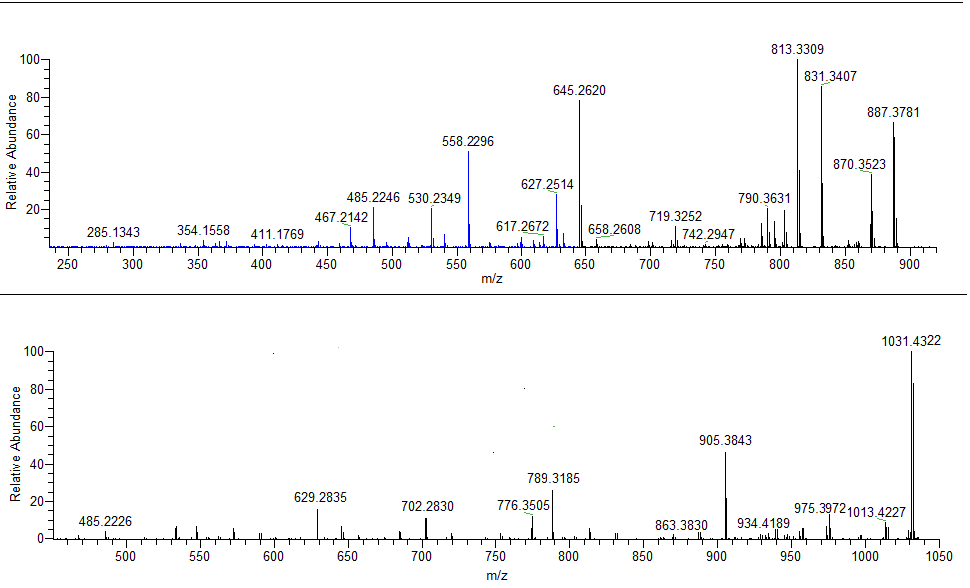


[M+H-NO]+

DASFH#SWG-NH2

DASFHSWG-NH2

b7 – H2O

ASFHS

FH

**[M+H]+ – H2O**

y4

y4

[M+H]+ – H2O

**y4**

**b6**

**b5**

**y5**

b7

**B**

**A**

**y7**

**b7**

b5

b6

y6

**y6**

y7

Supplement: Figure S3 — MS/MS of peptide [DASFHSWG-NH2] at m/z 905.3878 (A) and adduct [DASFHSWG-NH2+ NO] at m/z 1049.4487 (B). (DOCX) [file pone.0042053.s003.docx]

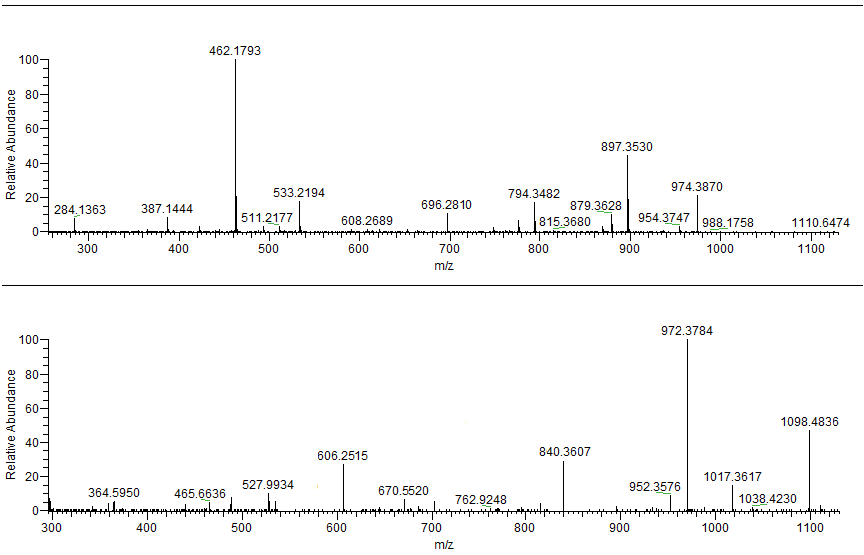


[M+H -NO]+

**[M+H]+ – H2O**

**y6**

y6

b4

y4

EFYAPWC#G

EFYAPWCG

b7

b6

b5

y5

**y4**

**A**

**B**

Supplement: Figure S4 — MS/MS of peptide [EFYAPWCG] at m/z 972.3920 (A) and adduct [EFYAPWCG + NO] at m/z 1116.4995 (B). (DOCX) [file pone.0042053.s004.docx]

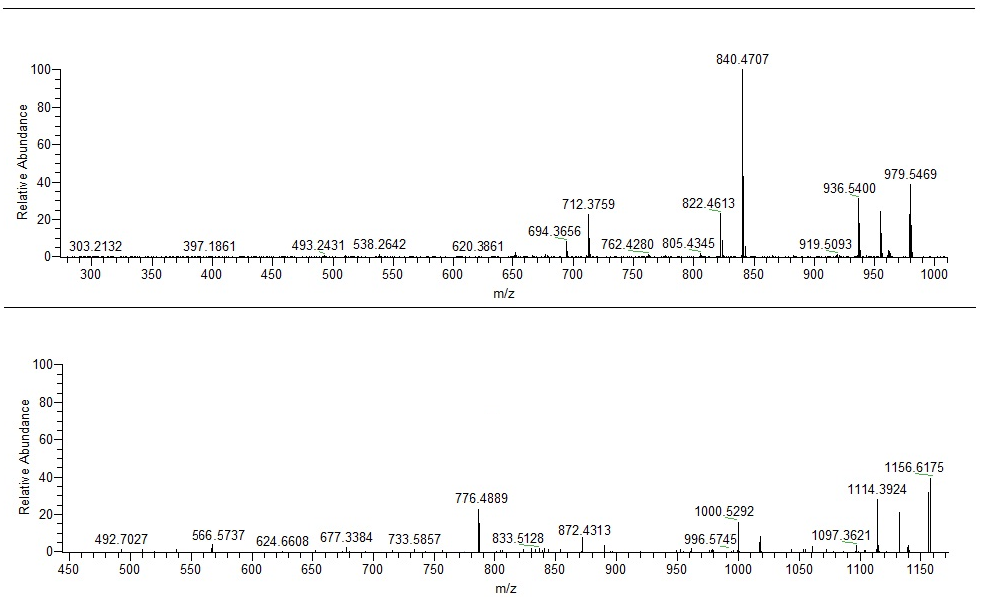


y7

y6

**b7**

[M+H-NO]+

**b6**

**[M+H]+ – H2O**

[M+H]+ – NH3

b7

b7 + H2O

b5

b6 + H2O

y2

YѰGGFLRKR

YGGFLRKR

b6

**B**

**A**

**B**

Supplement: Figure S5 — MS/MS of peptide [YGGFLRKR] at m/z 996.5738 (A) and adduct [YGGFLRKR + NDO] at m/z 1174.6365 (B). (DOCX) [file pone.0042053.s005.docx]

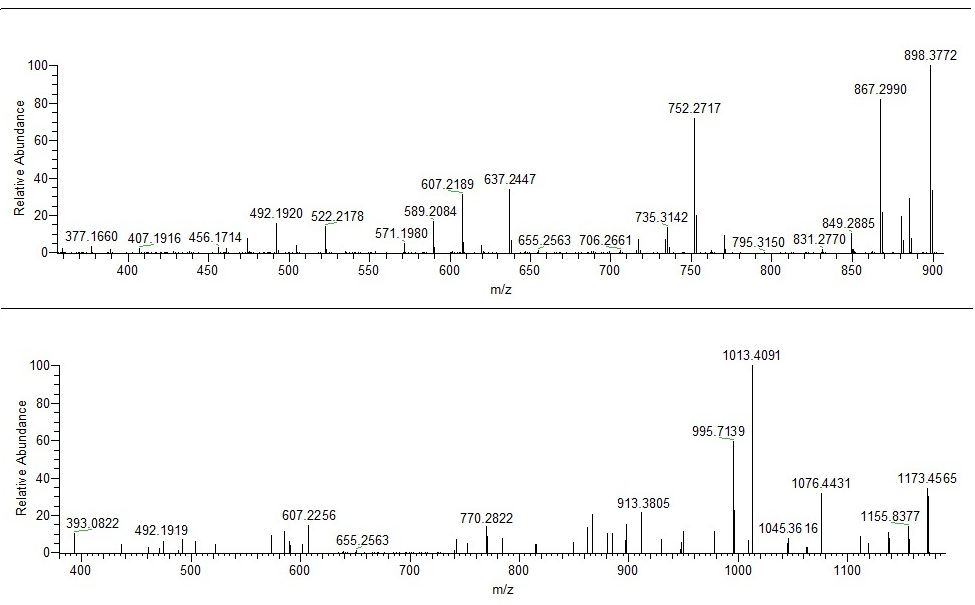


[M+H - NDO]+

y5

y4

**y7**

DYKѰDDDDK

**[M+H]+ – H2O**

DYKDDDDK

**b7**

y7

b7

b6

b5

b4

b3

y3

y6

y5

**y6**

y4

**B**

**A**

**B**

Supplement: Figure S6 — MS/MS of peptide [DYKDDDDK] at m/z 1013.4058 (A) and adduct [DYKDDDDK + NDO] at m/z 1191.4680 (B). (DOCX) [file pone.0042053.s006.docx]

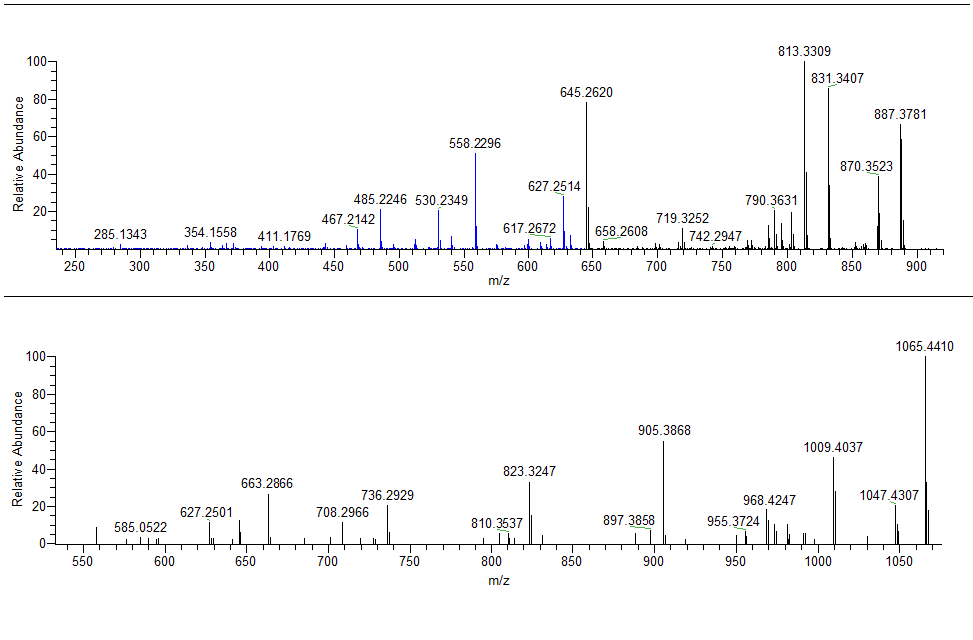


[M+H-NDO]+

**y6**

b7 – H2O

[M+H]+ – H2O

b7

DASFHѰSWG-NH2

DASFHSWG-NH2

**[M+H]+ – H2O**

**b7**

**y7**

**b6**

**y5**

**y4**

**b5**

**B**

**A**

y7

b6

b5

y6

y4

Supplement: Figure S7 — MS/MS of peptide [DASFHSWG-NH2] at m/z 905.3900 (A) and adduct [DASFHSWG-NH2+ NDO] at m/z 1083.4537 (B). (DOCX) [file pone.0042053.s007.docx]

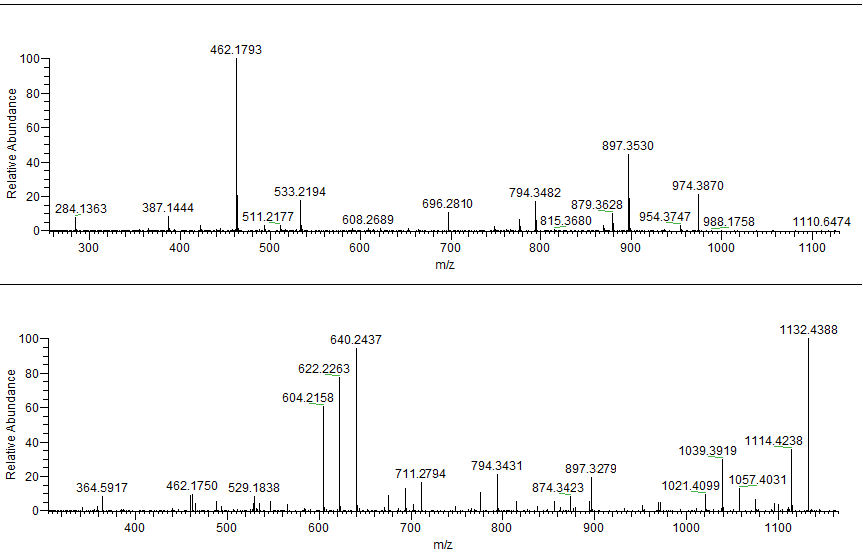


b6

b7

y4

**y6**

**y5**

PW

PWC

EFYAPWCѰG

EFYAPWCG

**y7**

**y4 – H2O**

**y4**

**[M+H]+ – H2O**

y5

b4

b6

b5

y6

**B**

**A**

Supplement: Figure S8 — MS/MS of peptide [EFYAPWCG] at m/z 972.3920 (A) and adduct [EFYAPWCG + NDO] at m/z 1150.4550 (B). (DOCX) [file pone.0042053.s008.docx]

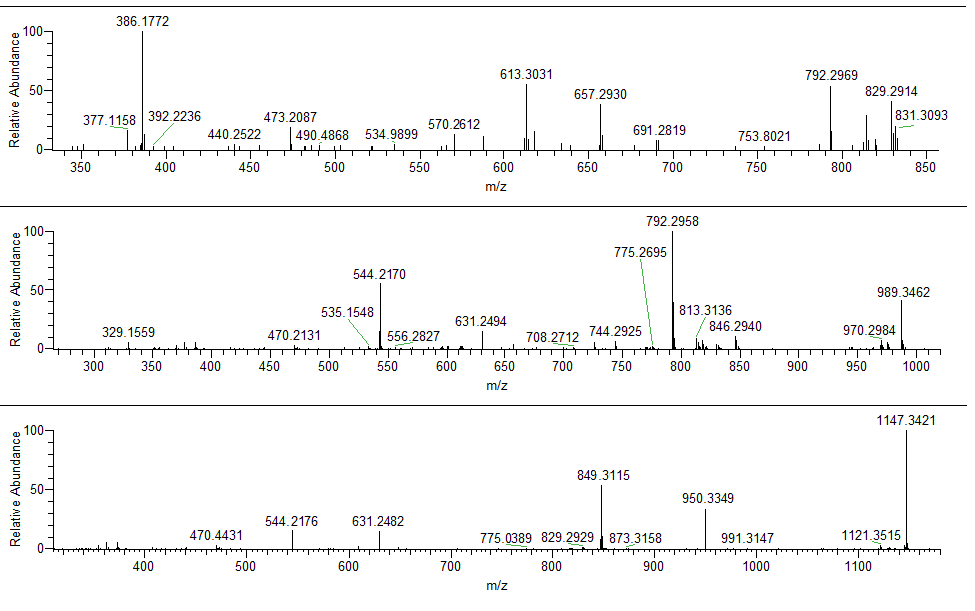


G†RGDSP†(?)C†

[M+H]+

**y6 + 1,2NQ**

**y6**

**y2**

G†RGDSPC†

GRGDSPC†

**[M+H]+ – H2O + 2(1,2NQ**)

**b4**

**b5**

**[M+H]+ \\2H+\\\**

**[M+H]+– H2O + 1,2NQ**

**b4**

**b5**

**B**

**C**

**A**

b6

**y6**

**[M+H]+ – H2O**

b5

b4

Supplement: Figure S9 — MS/MS of peptide [GRGDSPC +1,2NQ] monoadduct at m/z 849.3181 (A) and [GRGDSPC +1,2NQ] diadduct m/z 1007.3465 (B) and [GRGDSPC +1,2NQ] triadduct m/z 1165.3826 (C). (DOCX) [file pone.0042053.s009.docx]

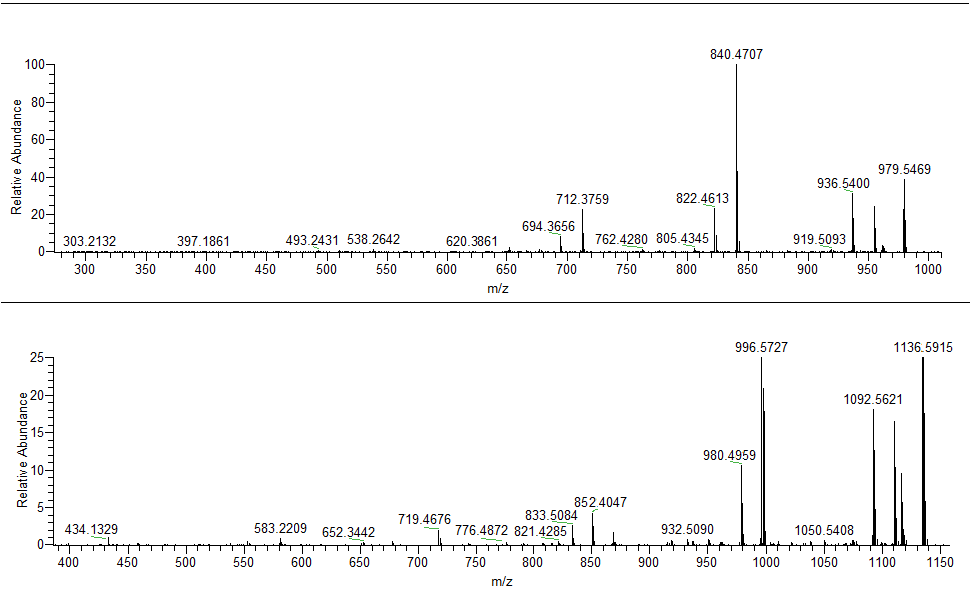


[M+H-1,2NQ]+

b7 + H2O

b5

b6 + H2O

y7

**b6**

**b7**

Y†GGFLRKR

YGGFLRKR

y6

[M+H]+ – NH3

b6

b7

y5

**B**

**A**

**b4**

**[M+H]+ – H2O**

y2

Supplement: Figure S10 — MS/MS of peptide [YGGFLRKR] at m/z 996.5738 (A) and adduct [YGGFLRKR +1,2NQ] at m/z 1154.6139 (B). (DOCX) [file pone.0042053.s010.docx]

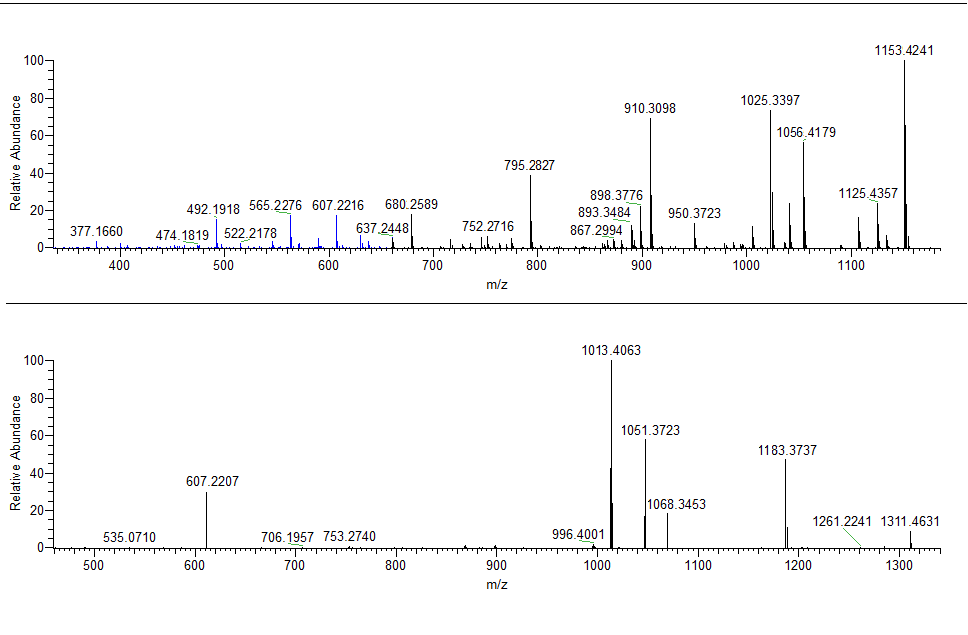


[M+H-1,2NQ]+

D†(?)YK†DDDDK

**b6 + 1,2NQ**

DYK†DDDDK

**b7 + 1,2NQ**

**b4**

y7

**[M+H]+ – H2O + 1,2NQ**

**y7**

y3

y4

**b7**

**b6**

**b3**

**b5**

y5

y5

**y6**

**B**

**A**

**[M+H]+ – H2O**

Supplement: Figure S11 — MS/MS of peptide [DYKDDDDK +1,2NQ] monoadduct at m/z 1171.4382 (A) and [DYKDDDDK +1,2NQ] diadduct at m/z 1329.4729 (B). (DOCX) [file pone.0042053.s011.docx]

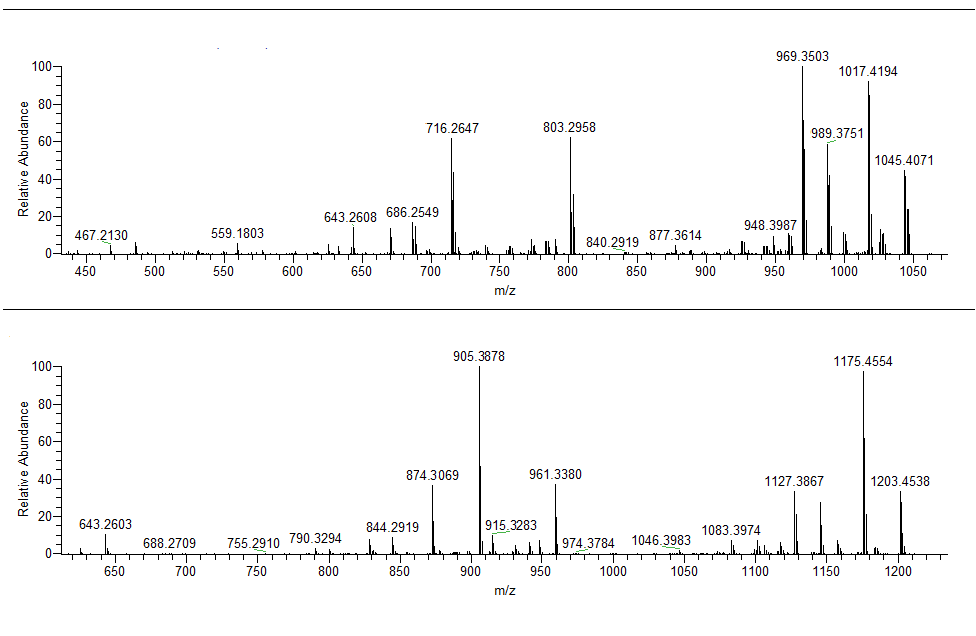


**b5 + 1,2NQ**

**b6 + 1,2NQ**

**b7**

**y4**

[M+H-1,2NQ]+

**[M+H]+ – H2O + 1,2NQ**

D†ASFH†SWG-NH2

DASFH†SWG-NH2

**[M+H]+ – H2O**

**y5**

**b6**

**y6**

**b5**

**y7**

**y4**

**B**

**A**

Supplement: Figure S12 — MS/MS of peptide [DASFHSWG-NH2+1,2NQ] monoadduct at m/z 1063.4248 (A) and [DASFHSWG-NH2+1,2NQ] diadduct at m/z 1221.4593 (B). (DOCX) [file pone.0042053.s012.docx]

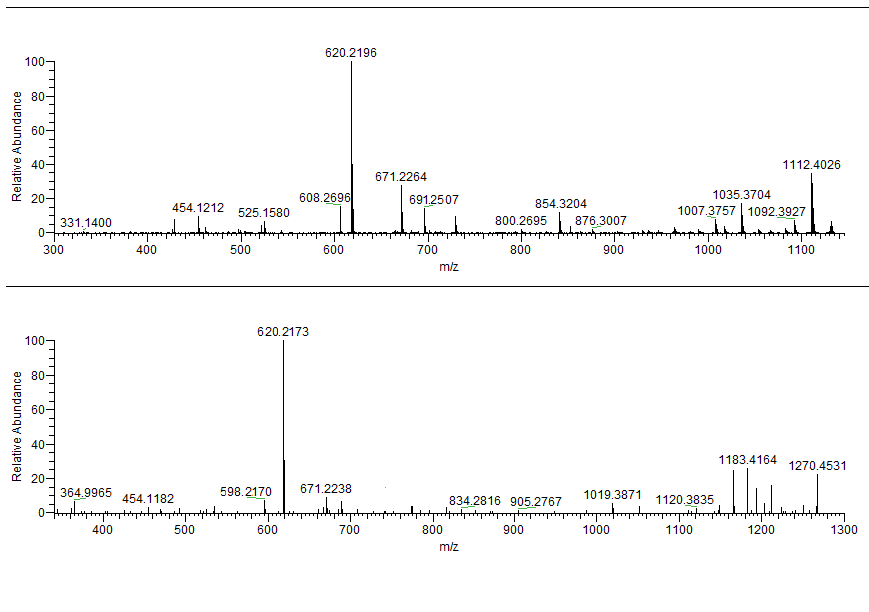


**[M+H]+ – H2O + 1,2NQ**

EFYAPWC††(?)G

**y4**

**y6**

b5

EFYAPWC†G

**y4**

**[M+H]+ – H2O**

**y5**

**A**

**B**

Supplement: Figure S13 — MS/MS of peptide [EFYAPWCG +1,2NQ] monoadduct at m/z 1130.4287 (A) and [EFYAPWCG +1,2NQ] diadduct at m/z 1288.4655 (B). (DOCX) [file pone.0042053.s013.docx]

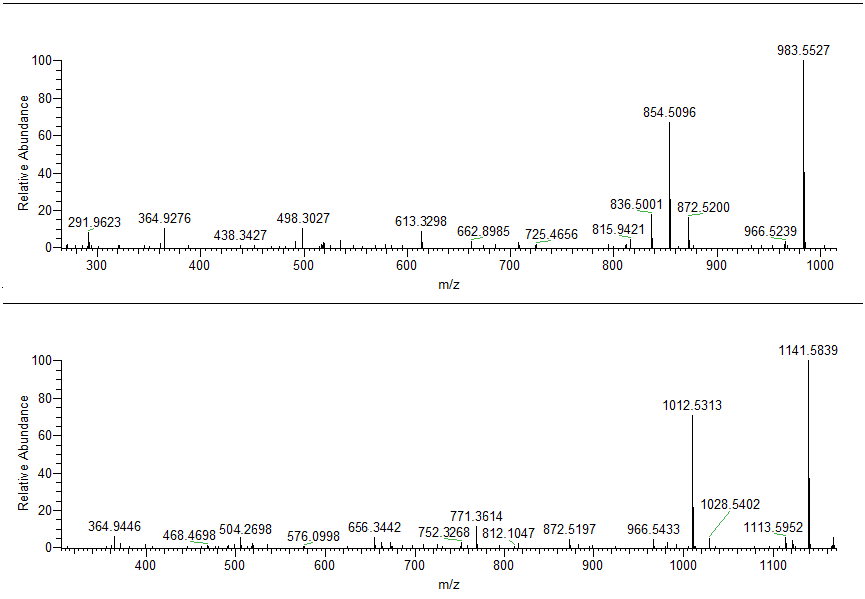


y7

[M+H]+ – H2O

y4

E†IVRDIKE

EIVRDIKE

y7

b5

**b7**

**[M+H]+– H2O**

**b5**

**b4**

b7

b4

**A**

**B**

Supplement: Figure S14 — MS/MS of peptide [EIVRDIKE] at m/z 1001.5626 (A) and adduct [EIVRDIKE +1,2NQ] at m/z 1159.5993 (B). (DOCX) [file pone.0042053.s014.docx]

**A**

**[M+H]+**

*
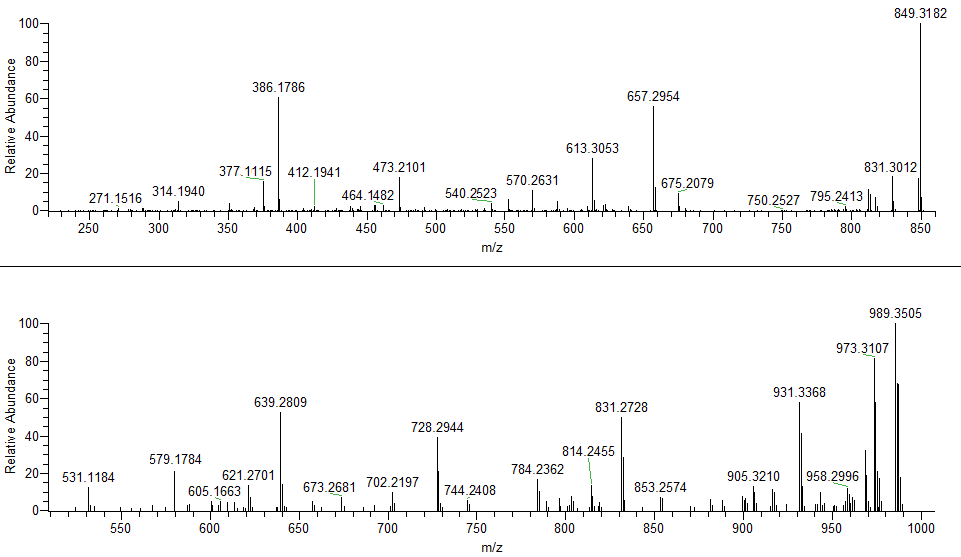
*

**[M+H]+ – H2O + 1,4NQ**

**[M+H]+ – H2O**

**y2**

**y3**

**b6**

G°RGDSPC°

b4

GRGDSPC°

b3

b6

b5

**B**

**[M+H]+ – H2O**

Supplement: Figure S15 — MS/MS of peptide [GRGDSPC +1,4NQ] monoadduct at m/z 849.3167 (A) and [GRGDSPC +1,4NQ] diadduct at m/z 1007.3466 (B). (DOCX) [file pone.0042053.s015.docx]

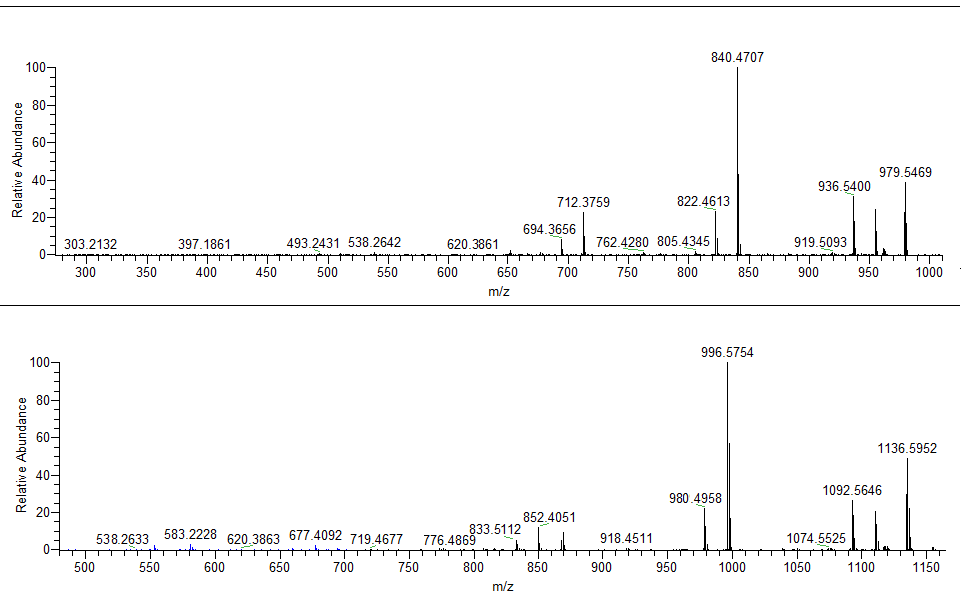


[M+H - 1,4NQ]+

y2

y5

b5

YGGFLRKR

Y°GGFLRKR

[M+H]+ – NH3

**B**

**A**

**[M+H]+ – H2O**

**b7**

**b4**

b6 + H2O

b7

b6

**b6**

b5

y6

b7 + H2O

y7

Supplement: Figure S16 — MS/MS of peptide [YGGFLRKR] at m/z 996.5738 (A) and adduct [YGGFLRKR +1,4NQ] at m/z 1154.6133 (B). (DOCX) [file pone.0042053.s016.docx]

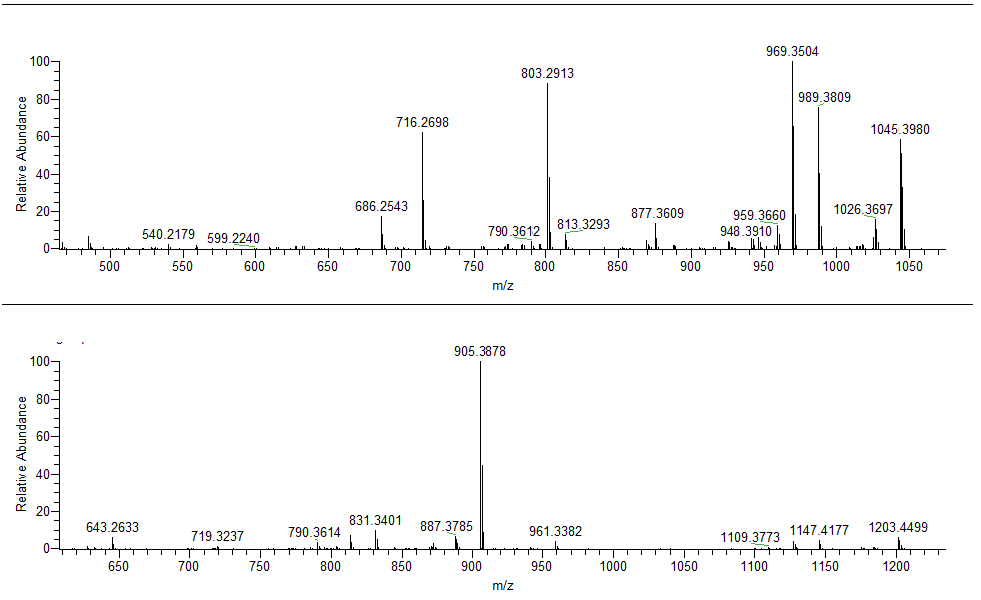


**b7 + 1,4NQ**

**b6 + 1,4NQ**

[M+H-1,4NQ]+

**[M+H]+ – H2O**

D°ASFH°SWG-NH2

**y6**

**y5**

**b7**

**b6**

DASFH°SWG-NH2

**y4**

**B**

**A**

**b5**

**y7**

**[M+H]+ – H2O + 1,4NQ**

Supplement: Figure S17 — MS/MS of peptide [DASFHSWG +1,4NQ] monoadduct at m/z 1063.4237 (A) and [DASFHSWG +1,4NQ] diadduct at m/z 1221.4593 (B). (DOCX) [file pone.0042053.s017.docx]

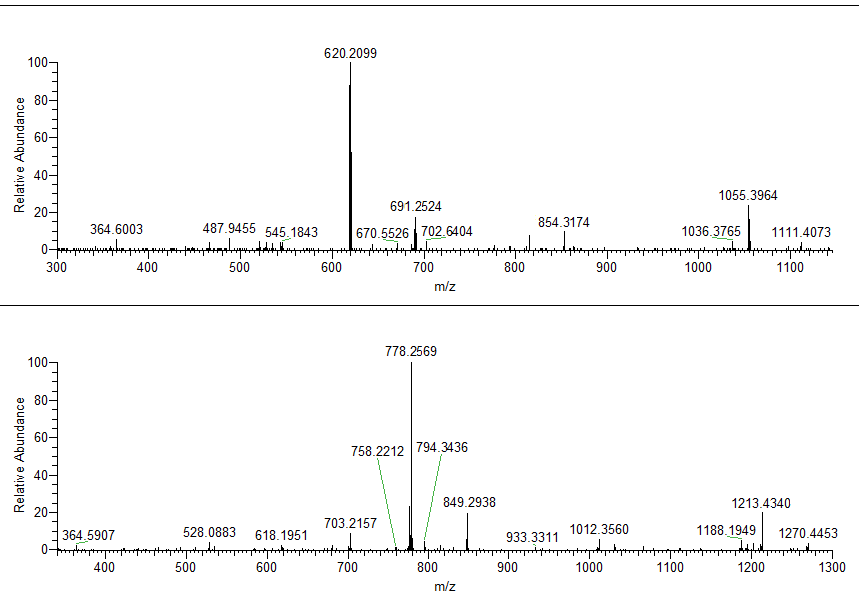


**y5 + 1,4NQ**

**y4 + 1,4NQ**

**y6­ + 1,4NQ**

EFYAPWC°°G

EFYAPWC°G

**b7 + 1,4NQ**

**y6**

**y5**

b6

**A**

**B**

**b7**

**y4**

Supplement: Figure S18 — MS/MS of peptide [EFYAPWCG +1,4NQ] monoadduct at m/z 1130.4287 (A) and [EFYAPWCG +1,4NQ] diadduct at m/z 1288.4655 (B). (DOCX) [file pone.0042053.s018.docx]

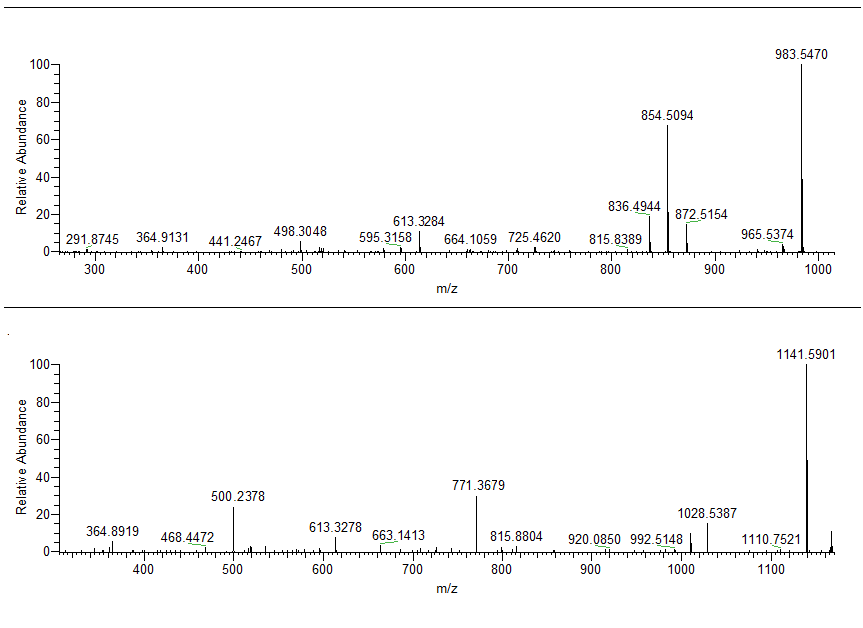


E°IVRDIKE

[M+H]+ – H2O

b7

EIVRDIKE

b7 – H2O

b4

b5

**b5**

**b35**

**[M+H]+ – H2O**

**B**

**A**

Supplement: Figure S19 — MS/MS of peptide [EIVRDIKE] at m/z 1001.5626 (A) and adduct [EIVRDIKE +1,4NQ] at m/z 1159.5993 (B). (DOCX) [file pone.0042053.s019.docx]
