# Supplementary material for: Characterization of Model Peptide Adducts with Reactive Metabolites of Naphthalene by Mass Spectrometry
Source: PLoS One. 2012 Aug 3;7(8):e42053. doi: 10.1371/journal.pone.0042053 (PMC3411726; doi:10.1371/journal.pone.0042053)
Supplement: Table S1 — PPM errors of adduct ions observed. (DOC) [file pone.0042053.s023.doc]

Table S1: PPM errors of adduct ions observed.

| **Table 1: Predicted and Observed Masses of Adducts** | | | | | | | | | | | |
| --- | --- | --- | --- | --- | --- | --- | --- | --- | --- | --- | --- |
| **Gly-Arg-Gly-Asp-Ser-Pro-Cys (GRGDSPC)** | | | | | | **Asp-Ala-Ser-Phe-His-Ser-Trp-Gly-NH2 (DASFHSWG)** | | | | | |
|  |  | **pH 8.5** | | **pH 7.4** | |  |  | **pH 8.5** | | **pH 7.4** | |
| **Metabolite** | **Predicted [M*+H]^+^ m/z** | **Observed [M*+H]^+^ m/z** | **Error (ppm)** | **Observed [M*+H]^+^ m/z** | **Error (ppm)** | **Metabolite** | **Predicted [M*+H]^+^ m/z** | **Observed [M*+H]^+^ m/z** | **Error (ppm)** | **Observed [M*+H]^+^ m/z** | **Error (ppm)** |
| **Naphthalene Epoxide** | 835.3403 | 835.3408 | -0.6 | X | X | **Naphthalene Epoxide** | 1049.4475 | 1049.4526 | -4.9 | 1049.4545 | -6.6702 |
| **Naphthalene Diol Epoxide** | 869.3460 | 869.3483 | -2.6 | X | X | **Naphthalene Diol Epoxide** | 1083.4530 | 1083.4557 | -2.5 | 1083.4578 | -4.4303 |
| **1,2-Naphthoquinone** | 849.3198 | 849.3181 | +2.0 | 849.3191 | 0.8242 | **1,2-Naphthoquinone** | 1063.4268 | 1063.4248 | +1.9 | 1063.4218 | 4.7018 |
| **1,4-Naphthoquinone** | 849.3198 | 849.3167 | +3.7 | 849.3163 | 4.1209 | **1,4-Naphthoquinone** | 1063.4268 | 1063.4237 | +2.9 | 1063.4218 | 4.7018 |
|  |  |  |  |  |  |  |  |  |  |  |  |
| **Tyr-Gly-Gly-Phe-Leu-Arg-Lys-Arg (YGGFLRKR)** | | | | | | **Glu-Phe-Tyr-Ala-Pro-Trp-Cys-Gly (EFYAPWCG)** | | | | | |
|  |  | **pH 8.5** | | **pH 7.4** | |  |  | **pH 8.5** | | **pH 7.4** | |
| **Metabolite** | **Predicted [M*+H]^+^ m/z** | **Observed [M*+H]^+^ m/z** | **Error (ppm)** | **Observed [M*+H]^+^ m/z** | **Error (ppm)** | **Metabolite** | **Predicted [M*+H]^+^ m/z** | **Observed [M*+H]^+^ m/z** | **Error (ppm)** | **Observed [M*+H]^+^ m/z** | **Error (ppm)** |
| **Naphthalene Epoxide** | 1140.6315 | X | X | X | X | **Naphthalene Epoxide** | 1116.4995 | 1116.4944 | +4.6 | 1116.4977 | 1.6122 |
| **Naphthalene Diol Epoxide** | 1174.6370 | 1174.6429 | -5.0 | X | X | **Naphthalene Diol Epoxide** | 1150.4550 | 1150.4575 | -2.2 | 1150.4581 | -2.6946 |
| **1,2-Naphthoquinone** | 1154.6108 | 1154.6139 | -2.7 | 1154.6116 | -0.6929 | **1,2-Naphthoquinone** | 1130.4287 | 1130.4261 | +2.3 | 1130.4301 | -1.2385 |
| **1,4-Naphthoquinone** | 1154.6108 | 1154.6133 | -2.2 | 1154.6074 | 2.9447 | **1,4-Naphthoquinone** | 1130.4287 | 1130.4234 | +4.7 | 1130.428 | 0.6192 |
|  |  |  |  |  |  |  |  |  |  |  |  |
| **Asp-Tyr-Lys-Asp-Asp-Asp-Asp-Lys (DYKDDDDK)** | | | | | | **Glu-Ile-Val-Arg-Asp-Ile-Lys-Glu (EIVRDIKE)** | | | | | |
|  |  | **pH 8.5** | | **pH 7.4** | |  |  | **pH 8.5** | | **pH 7.4** | |
| **Metabolite** | **Predicted [M*+H]^+^ m/z** | **Observed [M*+H]^+^ m/z** | **Error (ppm)** | **Observed [M*+H]^+^ m/z** | **Error (ppm)** | **Metabolite** | **Predicted [M*+H]^+^ m/z** | **Observed [M*+H]^+^ m/z** | **Error (ppm)** | **Observed [M*+H]^+^ m/z** | **Error (ppm)** |
| **Naphthalene Epoxide** | 1139.4482 | 1139.4521 | -3.4 | X | X | **Naphthalene Epoxide** | 1145.6201 | X | X | X | X |
| **Naphthalene Diol Epoxide** | 1191.4690 | 1191.4680 | +0.8 | X | X | **Naphthalene Diol Epoxide** | 1179.6256 | X | X | X | X |
| **1,2-Naphthoquinone** | 1171.4428 | 1171.4382 | +3.9 | 1171.4441 | 7.0853 | **1,2-Naphthoquinone** | 1159.5993 | 1159.5937 | +4.8 | 1159.5956 | 3.1908 |
| **1,4-Naphthoquinone** | 1171.4428 | 1171.4366 | +5.3 | 1171.4392 | 3.0731 | **1,4-Naphthoquinone** | 1159.5993 | 1159.6007 | -1.2 | 1159.5988 | 0.4312 |
