# Supplementary material for: Characterization of Model Peptide Adducts with Reactive Metabolites of Naphthalene by Mass Spectrometry
Source: PLoS One. 2012 Aug 3;7(8):e42053. doi: 10.1371/journal.pone.0042053 (PMC3411726; doi:10.1371/journal.pone.0042053)
Supplement: Supplementary file 3 [file pone.0042053.s020.doc]

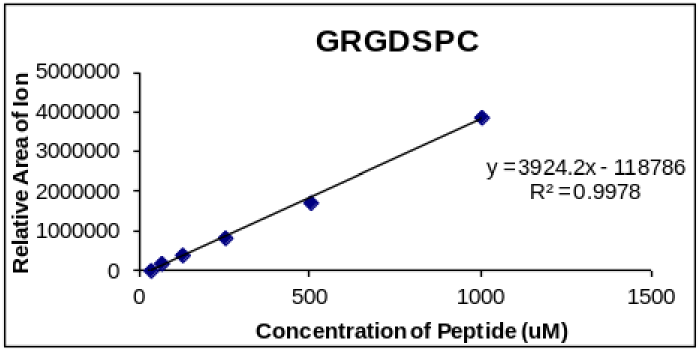
GRGDSPC
Relative Area of Ion
5000000
4000000
3000000
2000000
1000000
0
0
500
1000
1500
Concentration of Peptide (uM)
y = 3924.2x - 118786
R² = 0.9978

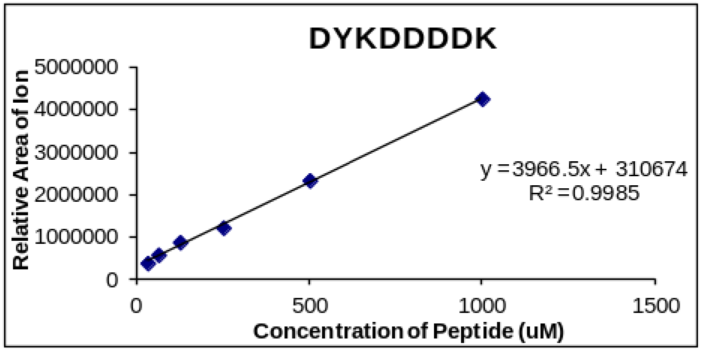
DYKDDDDK
Relative Area of Ion
5000000
4000000
3000000
2000000
1000000
0
0
500
1000
1500
Concentration of Peptide (uM)
y = 3966.5x + 310674
R² = 0.9985

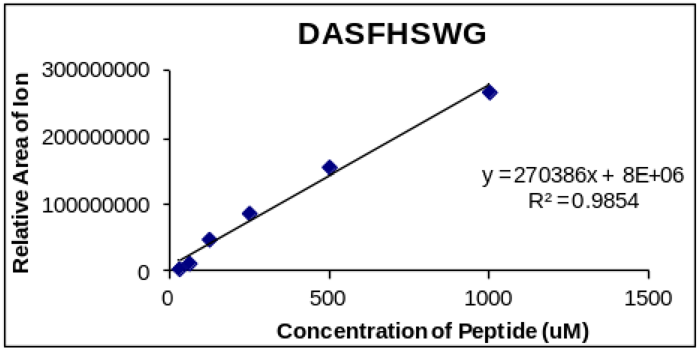
DASFHSWG
Relative Area of Ion
300000000
200000000
100000000
0
0
500
1000
1500
Concentration of Peptide (uM)
y = 270386x + 8E+06
R² = 0.9854
