# Supplementary material for: Characterization of Model Peptide Adducts with Reactive Metabolites of Naphthalene by Mass Spectrometry
Source: PLoS One. 2012 Aug 3;7(8):e42053. doi: 10.1371/journal.pone.0042053 (PMC3411726; doi:10.1371/journal.pone.0042053)
Supplement: Supplementary file 4 [file pone.0042053.s021.doc]

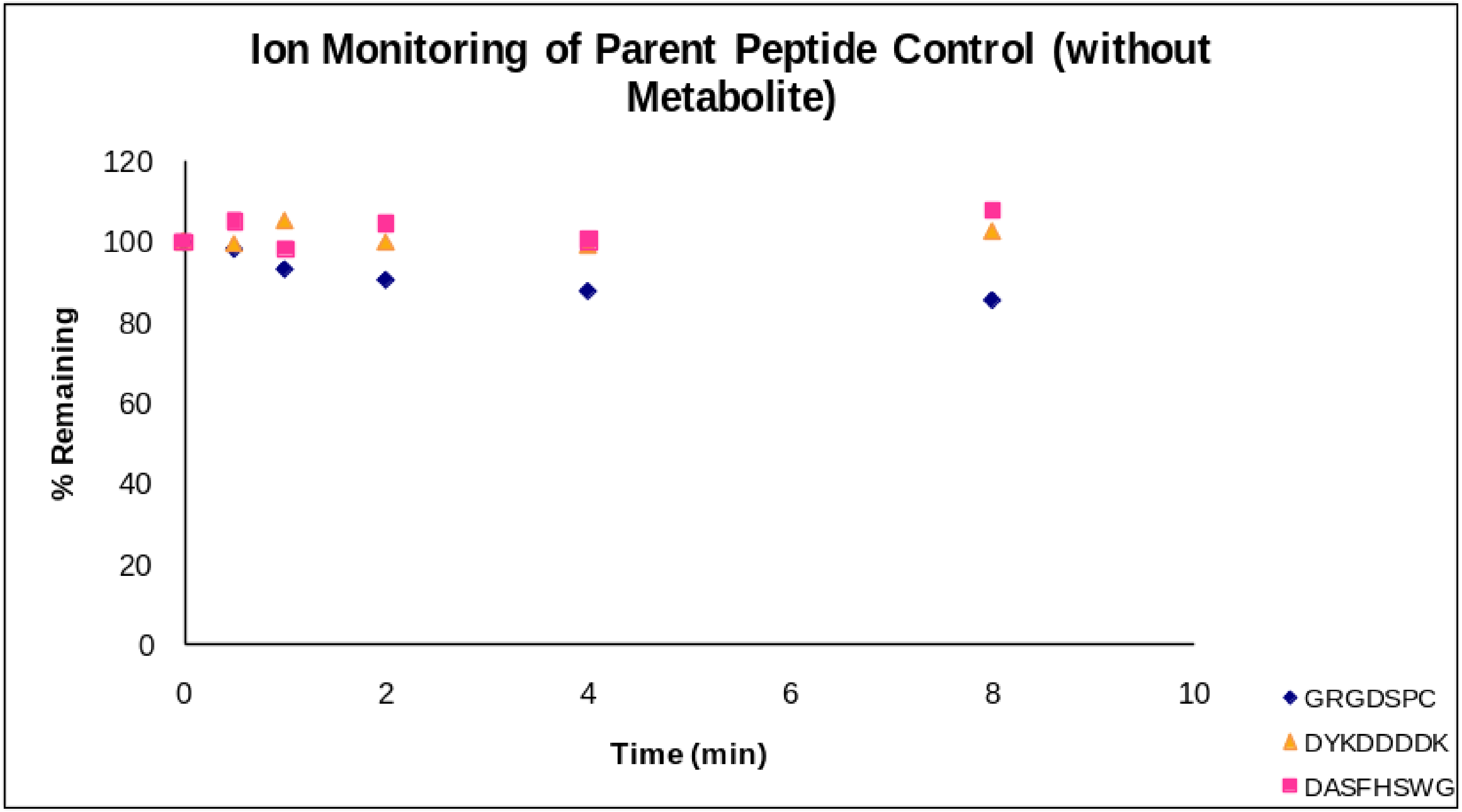
Ion Monitoring of Parent Peptide Control (without Metabolite)
% Remaining
120
100
80
60
40
20
0
0
2
4
6
8
10
Time (min)
GRGDSPC
DYKDDDDK
DASFHSWG
