# Supplementary material for: Characterization of Model Peptide Adducts with Reactive Metabolites of Naphthalene by Mass Spectrometry
Source: PLoS One. 2012 Aug 3;7(8):e42053. doi: 10.1371/journal.pone.0042053 (PMC3411726; doi:10.1371/journal.pone.0042053)
Supplement: Supplementary file 5 [file pone.0042053.s022.doc]

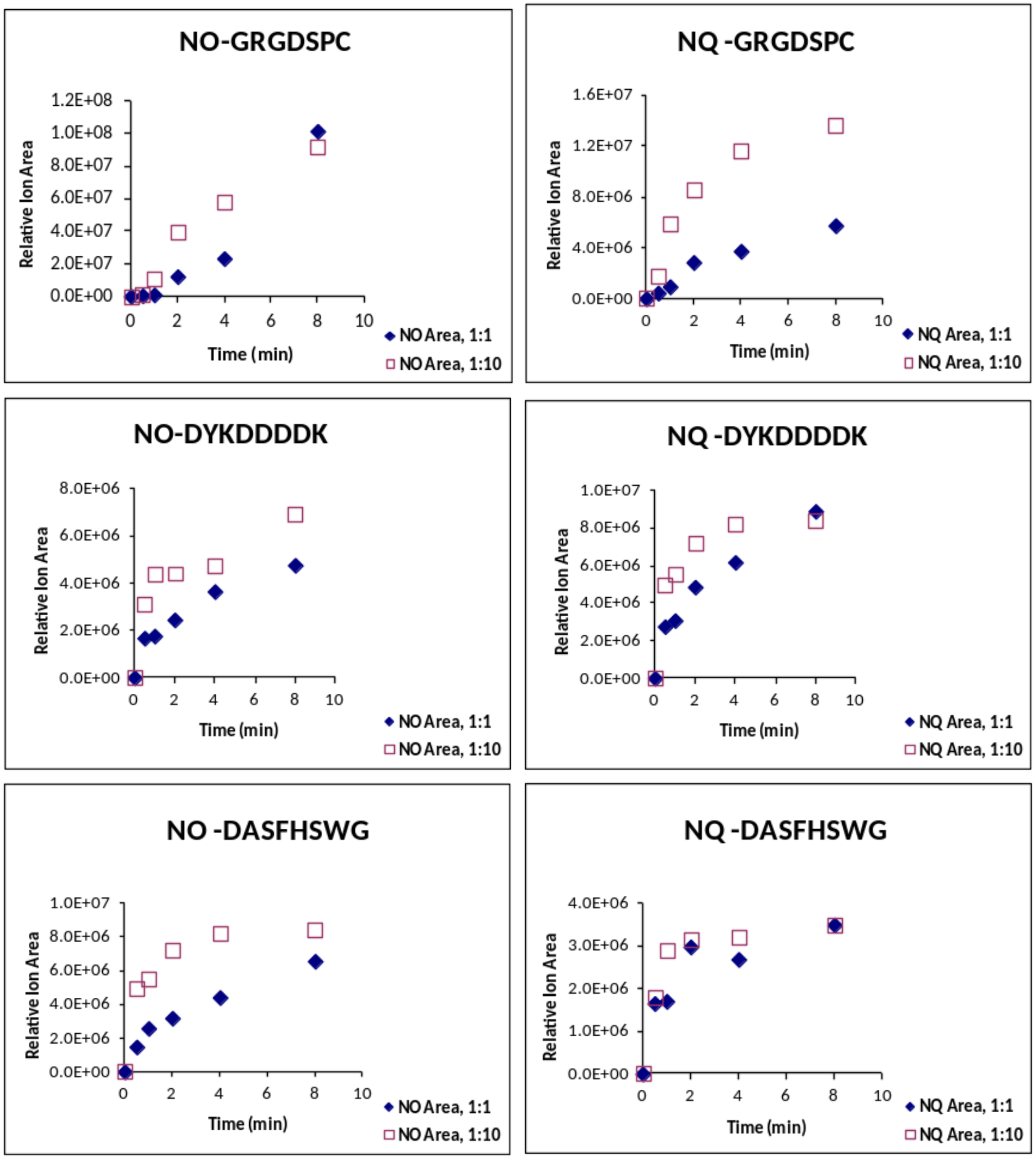
NO-GRGDSPC
Relative Ion Area
1.2E+08
1.0E+08
8.0E+07
6.0E+07
4.0E+07
2.0E+07
0.0E+00
0
2
4
6
8
10
Time (min)
NO Area, 1:1
NO Area, 1:10
NQ -GRGDSPC
Relative Ion Area
1.6E+07
1.2E+07
8.0E+06
4.0E+06
0.0E+00
Time (min)
NQ Area, 1:1
NQ Area, 1:10
NO-DYKDDDDK
Relative Ion Area
8.0E+06
6.0E+06
4.0E+06
2.0E+06
0.0E+00
Time (min)
NO Area, 1:1
NO Area, 1:10
NQ -DYKDDDDK
Relative Ion Area
1.0E+07
8.0E+06
6.0E+06
4.0E+06
2.0E+06
0.0E+00
Time (min)
NQ Area, 1:1
NQ Area, 1:10
NO -DASFHSWG
Relative Ion Area
1.0E+07
8.0E+06
6.0E+06
4.0E+06
2.0E+06
0.0E+00
Time (min)
NO Area, 1:1
NO Area, 1:10
NQ -DASFHSWG
Relative Ion Area
4.0E+06
3.0E+06
2.0E+06
1.0E+06
0.0E+00
Time (min)
NQ Area, 1:1
NQ Area, 1:10
